# Estrogen metabolites increase nociceptor hyperactivity in a mouse model of uterine pain

**DOI:** 10.1172/jci.insight.149107

**Published:** 2022-05-23

**Authors:** Zili Xie, Jing Feng, Tao Cai, Ronald McCarthy, Mark D. Eschbach, Yuhui Wang, Yonghui Zhao, Zhihua Yi, Kaikai Zang, Yi Yuan, Xueming Hu, Fengxian Li, Qin Liu, Aditi Das, Sarah K. England, Hongzhen Hu

**Affiliations:** 1 Center for the Study of Itch and Sensory Disorders and; 2 Department of Anesthesiology, Washington University School of Medicine, St. Louis, Missouri, USA.; 3 Center for Neurological and Psychiatric Research and Drug Discovery, Shanghai Institute of Materia Medica, Chinese Academy of Science, Shanghai, China.; 4 The First Affiliated Hospital of Chongqing Medical University, Chongqing, China.; 5 Center for Reproductive Health Sciences, Department of Obstetrics & Gynecology, Washington University School of Medicine, St. Louis, Missouri, USA.; 6 Department of Bioengineering, Neuroscience Program, University of Illinois Urbana-Champaign, Urbana, Illinois, USA.; 7 Department of Anesthesiology, Plastic Surgery Hospital, Chinese Academy of Medical Sciences and Peking Union Medical College, Beijing, China.; 8 Department of Nursing, Medical College of Nanchang University, Nanchang, China.

**Keywords:** Neuroscience, Ion channels, Pain

## Abstract

Pain emanating from the female reproductive tract is notoriously difficult to treat, and the prevalence of transient pelvic pain has been placed as high as 70%–80% in women surveyed. Although sex hormones, especially estrogen, are thought to underlie enhanced pain perception in females, the underlying molecular and cellular mechanisms are not completely understood. Here, we showed that the pain-initiating TRPA1 channel was required for pain-related behaviors in a mouse model of estrogen-induced uterine pain in ovariectomized female mice. Surprisingly, 2- and 4-hydroxylated estrogen metabolites (2- and 4-HEMs) in the estrogen hydroxylation pathway, but not estrone, estradiol, or 16-HEMs, directly increased nociceptor hyperactivity through TRPA1 and TRPV1 channels, and picomolar concentrations of 2- and 4-hydroxylation estrone (2- or 4-OHE1) could sensitize TRPA1 channel function. Moreover, both TRPA1 and TRPV1 were expressed in uterine-innervating primary nociceptors, and their expression was increased in the estrogen-induced uterine pain model. Importantly, pretreatment with 2- or 4-OHE1 recapitulated estrogen-induced uterine pain-like behaviors, and intraplantar injections of 2- and 4-OHE1 directly produced a TRPA1-dependent mechanical hypersensitivity. Our findings demonstrated that TRPA1 is critically involved in estrogen-induced uterine pain-like behaviors, which may provide a potential drug target for treating female reproductive tract pain.

## Introduction

Chronic pain caused by inflammation and nerve injury is a debilitating and persistent condition without universally effective treatment (1). Pain is a complex and multifactorial trait with tremendous interindividual variation (2–5), and women have greater pain sensitivity to many forms of clinical and experimentally induced pain responses, including pressure, heat, and chemical irritants (6–8). Clinically, women have higher rates of chronic pain conditions, such as migraine, arthritis, and fibromyalgia (9–12), and some chronic pelvic pain symptoms only occur in women, such as dysmenorrhea and endometriosis (13).

Sex steroid hormones, including progesterone, testosterone, and estrogen, have been linked to pain perception in clinical and preclinical studies. It has been widely accepted that testosterone and progesterone have anti-pain effects; the effects of estrogen remain controversial (14, 15). For instance, Birgitta et al. showed that pain in women is significantly higher during the menstrual and premenstrual phases when compared with the midmenstrual and ovulatory phases, which is correlated with the estrogen level (16). In rats, estrogen levels are lower during diestrus (~15–20 pg/mL) and higher during proestrus and estrus (~40–60 pg/mL; refs. 17–19). Fischer et al. reported that the nociceptive behavior of female rats in proestrus was significantly lower than that in the diestrus phase (20). On the other hand, numerous studies have demonstrated that estrogen promoted pain, for instance, estrogen was reported to exacerbate trigeminal nociceptive pain through upregulation of TRPV1 and ANO1 channel expression (21). In addition, Ji et al. reported that the visceromotor response fluctuated with the menstrual cycle, and the intensities of colorectal distensions were significantly higher in the proestrus phase when estrogen levels are higher (22). Moreover, chronic estrogen treatment was found to sensitize a subset of high-threshold mechanosensitive afferents innervating the uterine cervix, which might be responsible for increased pain responses produced by cervical distension (23). However, the molecular and cellular mechanisms underlying estrogen-mediated regulation of primary nociceptor activity remain elusive.

Primary sensory neurons in the dorsal root ganglion (DRG) and trigeminal ganglion sense noxious stimuli to initiate sensory hypersensitivity, such as pain and itch, the warning signs to protect from further tissue damage (24, 25). Transient receptor potential (TRP) channels are molecular sensors in the peripheral sensory nerve endings and contribute to nociceptive transmission by promoting action potential firing and regulating neurotransmitter release (26). Among them, TRPA1 and TRPV1 are selectively expressed by the small-sized nociceptors that are activated by noxious stimuli to produce either acute or persistent pain (27). Moreover, chronic estrogen treatment increased the excitability of sensory neurons and exacerbated inflammation-induced sensitization of sensory neurons by decreasing rheobase and the threshold of action potentials, thus promoting spontaneous action potential firing (28), suggesting that estrogen might directly regulate nociceptor excitability. However, the molecular basis of estrogen regulation of nociceptor excitability is not understood.

Here, we showed that although estrogen did not directly affect nociceptor activity, estrogen metabolites in the 2- and 4-hydroxylation pathways promoted nociceptor excitability through activation/sensitization of the nociceptive ion channels TRPA1 and TRPV1. Importantly, we showed that TRPA1 mediated not only acute pain responses evoked by 2- and 4-hydroxylation estrone (2- and 4-OHE1) but also visceral hypersensitivity in mouse models of uterine pain induced by either chronic estrogen treatment or acute application of 2- or 4-OHE1. Our studies uncovered a role of TRPA1 in estrogen-related pain perception, advancing the understanding of the role of primary nociceptors in estrogen-induced pain and/or enhancement of pain perception.

## Results

### Chronic estrogen treatment causes uterine pain.

**Figure 1:**
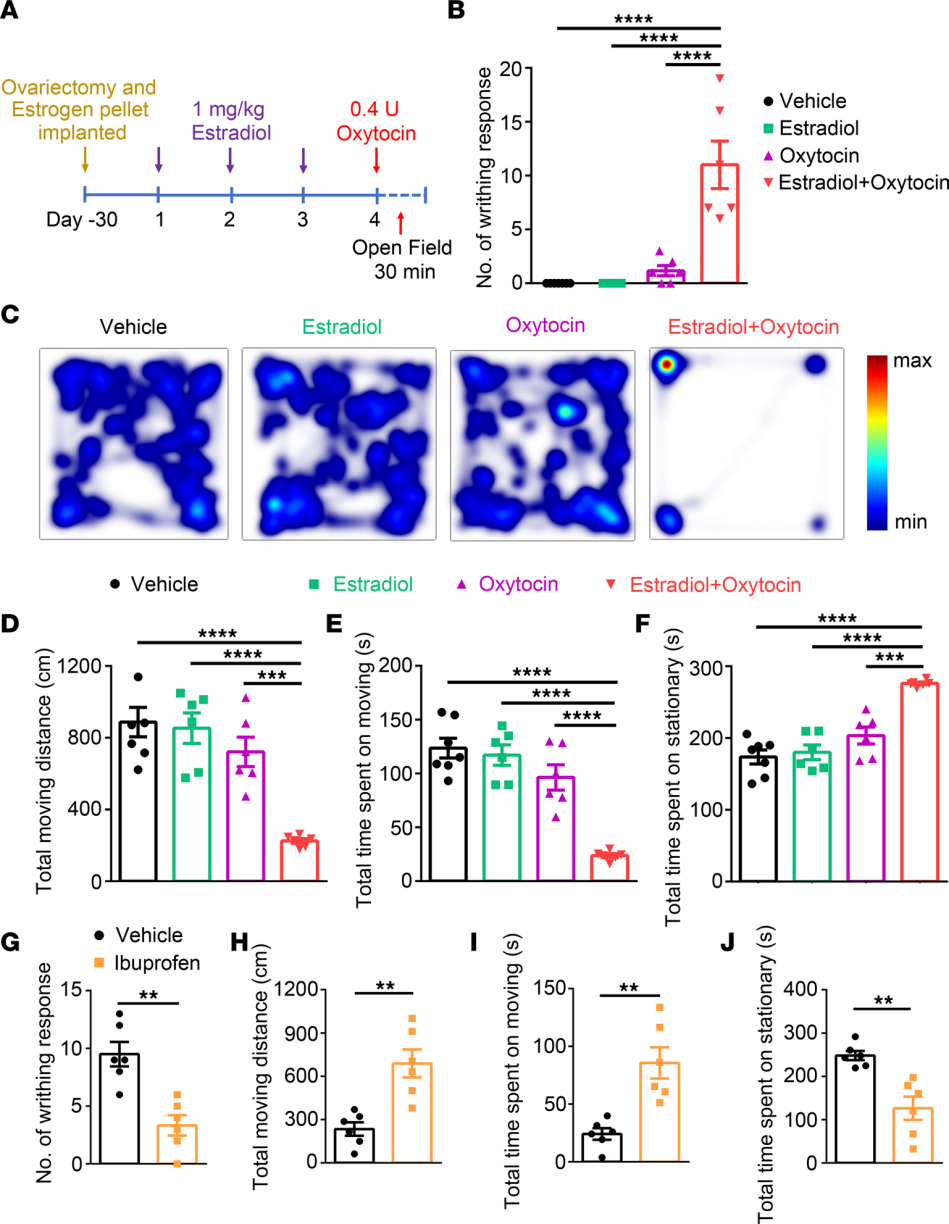
Pain-related responses in the mouse model of uterine pain. (**A**) Schematic diagram illustrates the protocol for generating a mouse model of uterine pain in ovariectomized female mice implanted with estrogen pellets. (**B**) Oxytocin-induced writhing response in vehicle group, estradiol group (pretreatment with estradiol benzoate without application of oxytocin), oxytocin group (application of oxytocin without pretreatment with estradiol benzoate) and estradiol + oxytocin group (pretreatment with estradiol benzoate followed by application of oxytocin). *n =* 6 mice per group, 1-way ANOVA. ****P < 0.0001. (**C**) Representative images illustrate heatmaps of voluntary movements of mice subjected to different treatments in open-field test in home cages. (**D**–**F**) Quantification of voluntary movements, including total moving distance (**D**), total time spent moving (**E**), and total time spent stationary (**F**), *n =* 6 mice per group, 1-way ANOVA. ***P < 0.001, ****P < 0.0001. (**G**–**J**) Ibuprofen significantly reduced the number of writhing responses (**G**) and total time spent stationary (**J**) and increased the total moving distance (**H**) and total time spent moving (**I**); *n =* 6 mice per group, unpaired Student’s *t* test. **P < 0.01. All data are expressed as mean ± SEM.

Although clinical evidence has clearly shown that women have higher pain sensitivity compared with men, likely due to a higher level of estrogen (29, 30), where and how estrogen acts to regulate pain sensitivity in women is not well understood. To address these questions, we generated a mouse model of uterine pain by administrating estradiol benzoate for 3 consecutive days followed by an i.p. injection of oxytocin at day 4 in ovariectomized WT C57BL/6J female mice implanted with estrogen pellets (Figure 1A). Although it is considered as a mouse model of primary dysmenorrhea (31, 32), this model also resembles human labor pain in which concentrations of estrogen and oxytocin are elevated (33, 34). As expected, oxytocin-induced uterus contraction markedly increased writhing response in estrogen-treated mice, whereas mice treated with vehicle, estradiol, or oxytocin alone rarely had a writhing response (Figure 1B). Of note, in the uterine pain model mice, the serum estradiol level was significantly increased to approximately 150 pg/mL compared with vehicle-treated mice (Supplemental Figure 1; supplemental material available online with this article; https://doi.org/10.1172/jci.insight.149107DS1), which is close to the serum estradiol level at the ovulation phase of the menstrual cycle in humans.

To quantify pain-like responses in the uterine pain model mice, we also measured voluntary movements, including the time mice spent moving or stationary and the total traveling distance around the cage, for the following reasons: (a) monitoring changes in voluntary movements in mice subjected to either inflammation or neuropathy is a simple, observer-independent, and more objective measure of the global level of pain response than reflexive measures and much more sensitive to analgesic drug effects (35); and (b) in a mouse model of acetic acid–induced visceral pain, monitoring changes in movement over time is a more sensitive parameter to identify differences in visceral nociception compared with writhing reflexes (36). Consistent with enhanced writhing response, total traveling distance and time spent moving were decreased and time spent stationary was increased in female mice treated with both estradiol benzoate and oxytocin compared with female mice treated with vehicle, estradiol, or oxytocin alone (Figure 1, C–F). In contrast, male mice treated with estradiol and oxytocin did not show any visceral pain-like behaviors (Supplemental Figure 2). Moreover, the pain-inhibiting NSAID, ibuprofen, markedly reduced the number of writhing responses (Figure 1G). Ibuprofen also increased the total traveling distance and the time spent moving but decreased the time spent stationary (Figure 1, H–J), confirming that pain-like behaviors were reliably produced in the uterine pain model mice.

### Estrogen metabolites but not estrogens or oxytocin directly activate DRG neurons.

To identify the cellular mechanisms underlying uterine pain response produced by estrogen and oxytocin, we used live-cell Ca^2+^ imaging to determine whether oxytocin directly activates dissociated DRG neurons. Surprisingly, no effect of oxytocin was observed even with the highest concentration applied (30 μM; Supplemental Figure 3A), which is in marked contrast to a recent study showing that 10 μM oxytocin elicited a robust intracellular Ca^2+^ ([Ca^2+^]_i_) response in DRG neurons isolated from male and female WT mice (37). Our result suggests that oxytocin likely causes pain by inducing uterine contractions rather than directly sensitizing nociceptors, which is also supported by the finding that male mice subjected to the same protocol did not develop overt visceral pain responses (Supplemental Figure 2 and ref. 32). Moreover, when 100 μM estrogen, including estrone (E1) and estradiol (E2), were applied to DRG neurons, no changes in [Ca^2+^]_i_ were observed (Supplemental Figure 3, B and C). Together, these results suggest that neither oxytocin nor estrogens directly activate and/or sensitize nociceptors.

**Figure 2:**
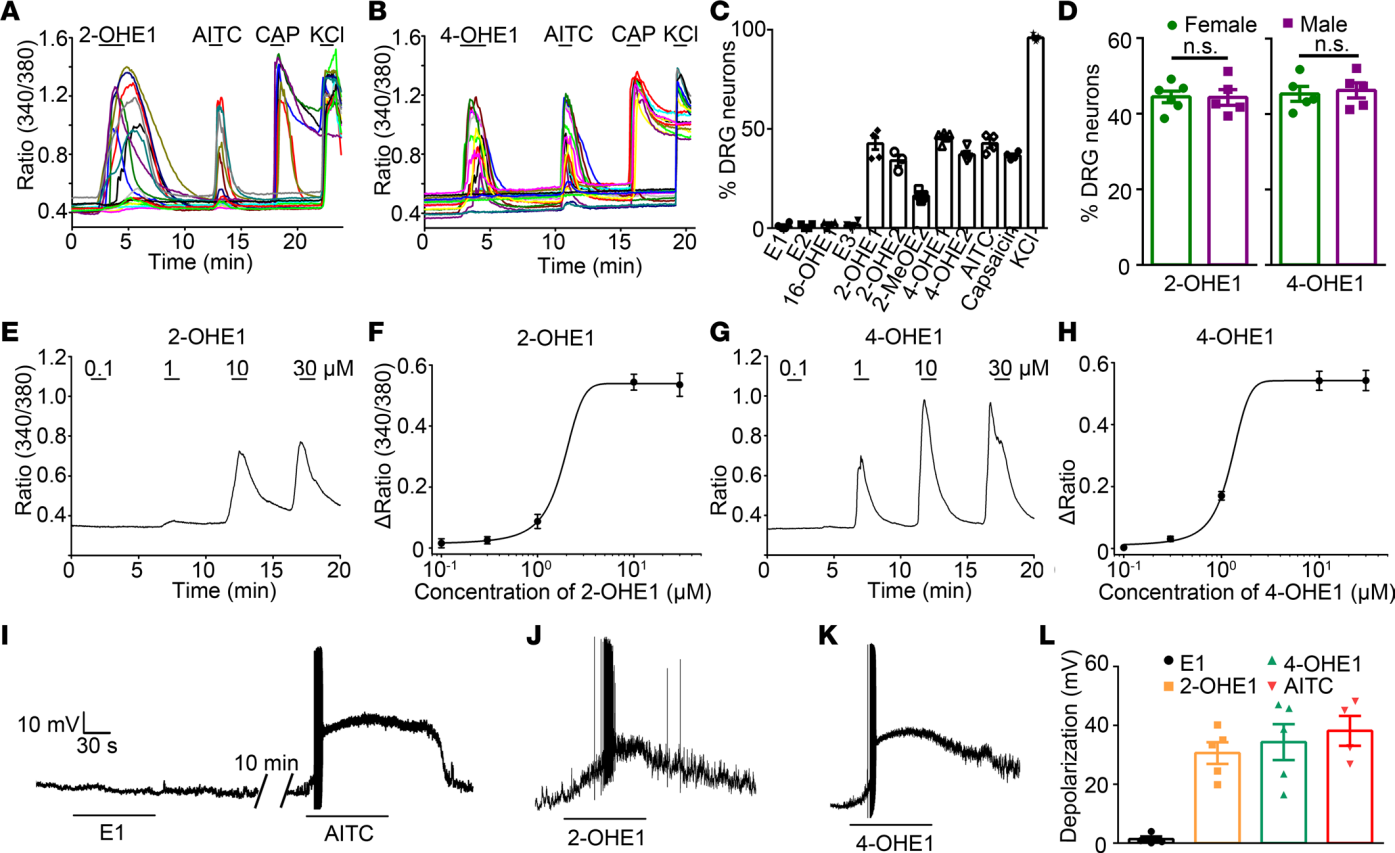
Estrogen metabolites 2- and 4-HEMs directly activate DRG neurons. (**A** and **B**) Representative time-lapse traces of 2-OHE1– (**A**) and 4-OHE1–induced (**B**) [Ca^2+^]_i_ responses in DRG neurons from WT mice. (**C**) Percentage of DRG neurons responding to estrogen and its metabolites. *n =* 5 mice (>200 neurons each). (**D**) Percentage of DRG neurons isolated from female and male mice responding to 2- and 4-OHE1. *n =* 5 to 6 coverslips per group from 4 mice (>150 neurons each). (**E**–**H**) Representative [Ca^2+^]_i_ responses and dose-response curves of [Ca^2+^]_i_ responses in DRG neurons from WT mice responding to different concentrations of 2-OHE1 (**E** and **F**) and 4-OHE1 (**G** and **H**). (**I**–**K**) Representative voltage traces show membrane potential depolarization and action potential firing in DRG neurons isolated from WT mice induced by E1 (**I**), 2-OHE1 (**J**), and 4-OHE1 (**K**). (**L**) Quantification of E1, 2-OHE1, 4-OHE1, and AITC-induced membrane potential depolarization in DRG neurons isolated from WT mice. *n =* 4 to 5 from 3 mice.

Notably, estrogen is primarily metabolized through 3 hydroxylation pathways in which hydroxylation at positions of C2, C4, and C16 converts them into catechol estrogens, including 2-hydroxylation estrone (2-OHE1) 2-hydroxyestradiol (2-OHE2), 2-methoxyestradiol (2MeOE2), 4-hydroxylation estrone (4-OHE1), 4-hydroxyestradiol (4-OHE2), 16-hydroxylation estrone (16-OHE1), and estriol (E3) (Supplemental Figure 4 and ref. 38). Surprisingly, 2- and 4-hydroxylated estrogen metabolites (2- and 4- HEMs) but not E1, E2, or 16-HEMs elicited a robust increase in [Ca^2+^]_i_ in WT DRG neurons, which also responded to capsaicin (Figure 2, A–C). Interestingly, DRG neurons isolated from male and female WT mice displayed comparable increases in [Ca^2+^]_i_ to both 2- and 4-OHE1 (Figure 2D), suggesting that there are no sex differences in TRPV1-expressing nociceptor sensitivity to HEMs. 2-OHE1 or 4-OHE1 increased [Ca^2+^]_i_ in DRG neurons in a concentration-dependent manner with EC_50_ values of 1.82 ± 0.10 and 1.24 ± 0.04 μM, respectively (Figure 2, E–H). Consistent with the Ca^2+^ imaging results, administration of 100 μM 2-OHE1 and 4-OHE1 but not E1 also evoked action potential firing and membrane depolarization in WT DRG neurons (Figure 2, I–L). These results suggest that 2- and 4-HEMs but not E1, E2, or 16-HEMs directly activate primary nociceptors in vitro.

### Estrogen metabolites are a new class of activators for TRPA1 and TRPV1.

**Figure 3:**
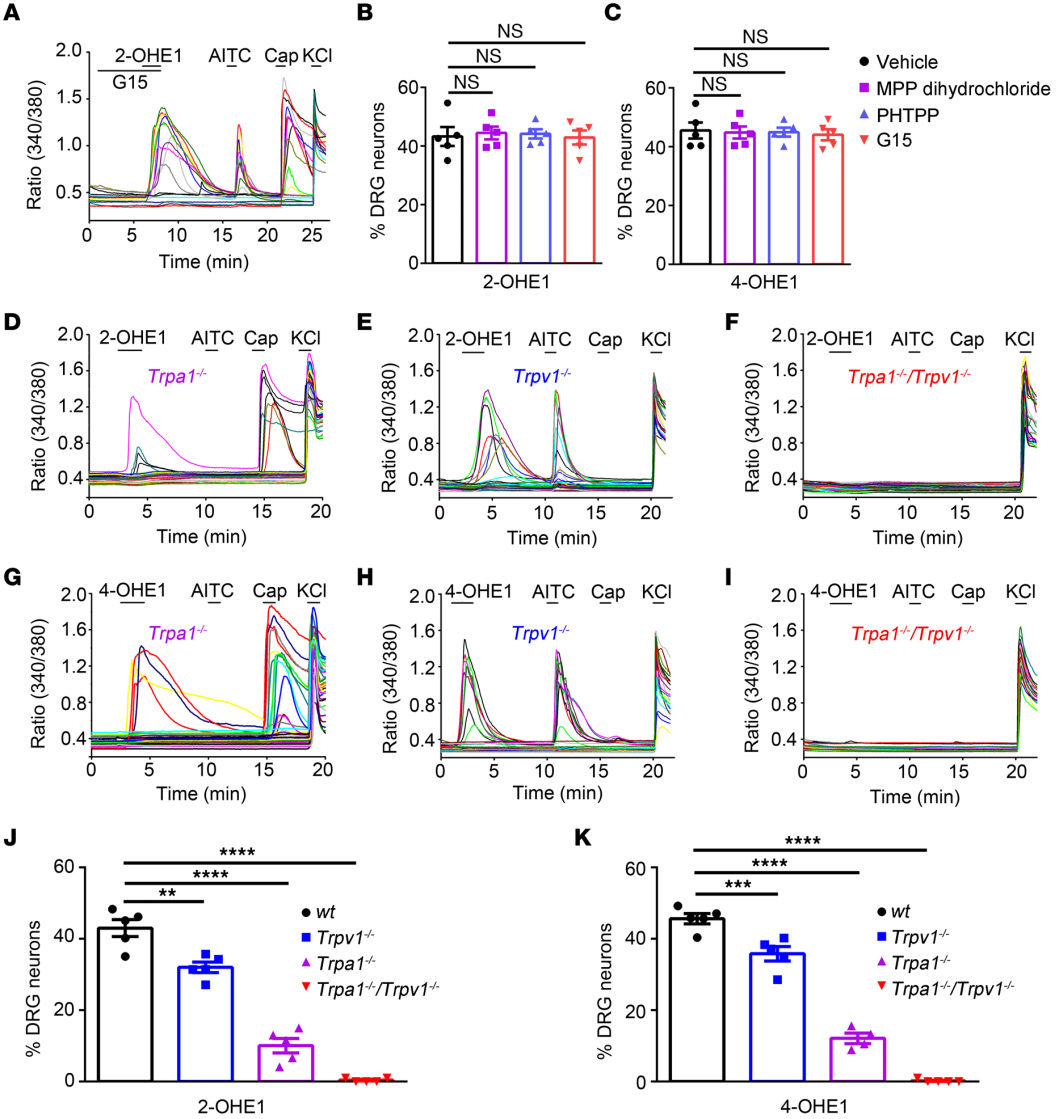
TRPA1 and TRPV1 are required for 2- and 4-OHE1–induced DRG activation. (**A**) Representative traces show that the GPER antagonist G15 had no effect on 2-OHE1–induced [Ca^2+^]_i_ responses in DRG neurons. (**B** and **C**) Summarized data show that estrogen receptor antagonists (at 1 μM) had no effect on 2-OHE1–induced [Ca^2+^]_i_ responses in DRG neurons. *n =* 5 coverslips from 3 mice per group (>150 neurons each). One-way ANOVA; NS, not significant. (**D**–**I**) Representative time-lapse traces of 2-OHE1– (**D**–**F**) and 4-OHE1–induced (**G**–**I**) [Ca^2+^]_i_ responses in the *Trpa1^−/−^*, *Trpv1^−/−^*, *Trpa1^−/−^Trpv1^−/−^* DRG neurons. (**J**–**K**) Percentage of DRG neurons responding to 2-OHE1 (**J**) and 4-OHE1 (**K**) in DRG neurons isolated from WT, *Trpa1^−/−^*, *Trpv1^−/−^*, and *Trpa1^−/−^Trpv1^−/−^* mice. *n =* 5 coverslips from 4 mice per group (>200 neurons each). **P < 0.01, ***P < 0.001, ****P < 0.0001, 1-way ANOVA.

We next tested whether estrogen receptors, including nuclear estrogen receptors (ERα and ERβ) and a G protein–coupled estrogen receptor 1 (GPER), mediate 2- and 4-OHE1–induced [Ca^2+^]_i_ responses in DRG neurons by using their specific inhibitors. All of these estrogen receptor inhibitors had no significant effect on the proportions of neurons responding to 2- or 4-OHE1 with [Ca^2+^]_i_ increase (Figure 3, A–C), suggesting that 2- and 4-HEM–elicited [Ca^2+^]_i_ responses in DRG neurons are estrogen receptor independent.

Since TRPA1 is a nociceptive ion channel that is activated by many different classes of chemical compounds, we next tested 2- and 4-OHE1–induced [Ca^2+^]_i_ responses in DRG neurons isolated from *Trpa1^–/–^* mice. Strikingly, the proportions of DRG neurons responding to 100 μM 2- and 4-OHE1 were substantially reduced in *Trpa1^–/–^* DRG neurons when compared with DRG neurons isolated from WT mice (Figure 3, D, G, J, and K), suggesting that sensory neuron–expressed TRPA1 is required for 2- and 4-OHE1–induced [Ca^2+^]_i_ responses. However, about 10% of DRG neurons isolated from *Trpa1^–/–^* mice could still be activated by 100 μM 2- and 4-OHE1 (Figure 3, D, G, J, and K), indicating that TRPA1 is not the sole target for 2- and 4-HEMs in DRG neurons.

Interestingly, all DRG neurons responding to 2- and 4-HEMs could also be activated by the TRPV1 agonist capsaicin (Figure 3A); therefore, we tested whether TRPV1 is also involved. Indeed, the proportions of DRG neurons responding to 100 μM 2- and 4-OHE1 were significantly reduced but not abolished in DRG neurons isolated from *Trpv1^–/–^* mice when compared with WT mice (Figure 3, E, H, J, and K). Remarkably, 2- and 4-OHE1–induced [Ca^2+^]_i_ responses were completely abolished in DRG neurons isolated from the *Trpa1^–/–^/ Trpv1^–/–^* double KO (dKO) mice (Figure 3, F, I–K), suggesting that both TRPA1 and TRPV1 are involved in [Ca^2+^]_i_ responses induced by 100 μM 2- and 4-OHE1. On the other hand, the [Ca^2+^]_i_ response evoked by a lower concentration of 2- and 4-OHE1 (10 μM) was absent in DRG neurons isolated from the *Trpa1^–/–^* mice but still present in DRG neurons from the *Trpv1^–/–^* and WT mice (Supplemental Figure 5), suggesting that 2- and 4-OHE1 activate only TRPA1 but not TRPV1 in primary nociceptors at concentrations lower than 10 μM.

**Figure 4:**
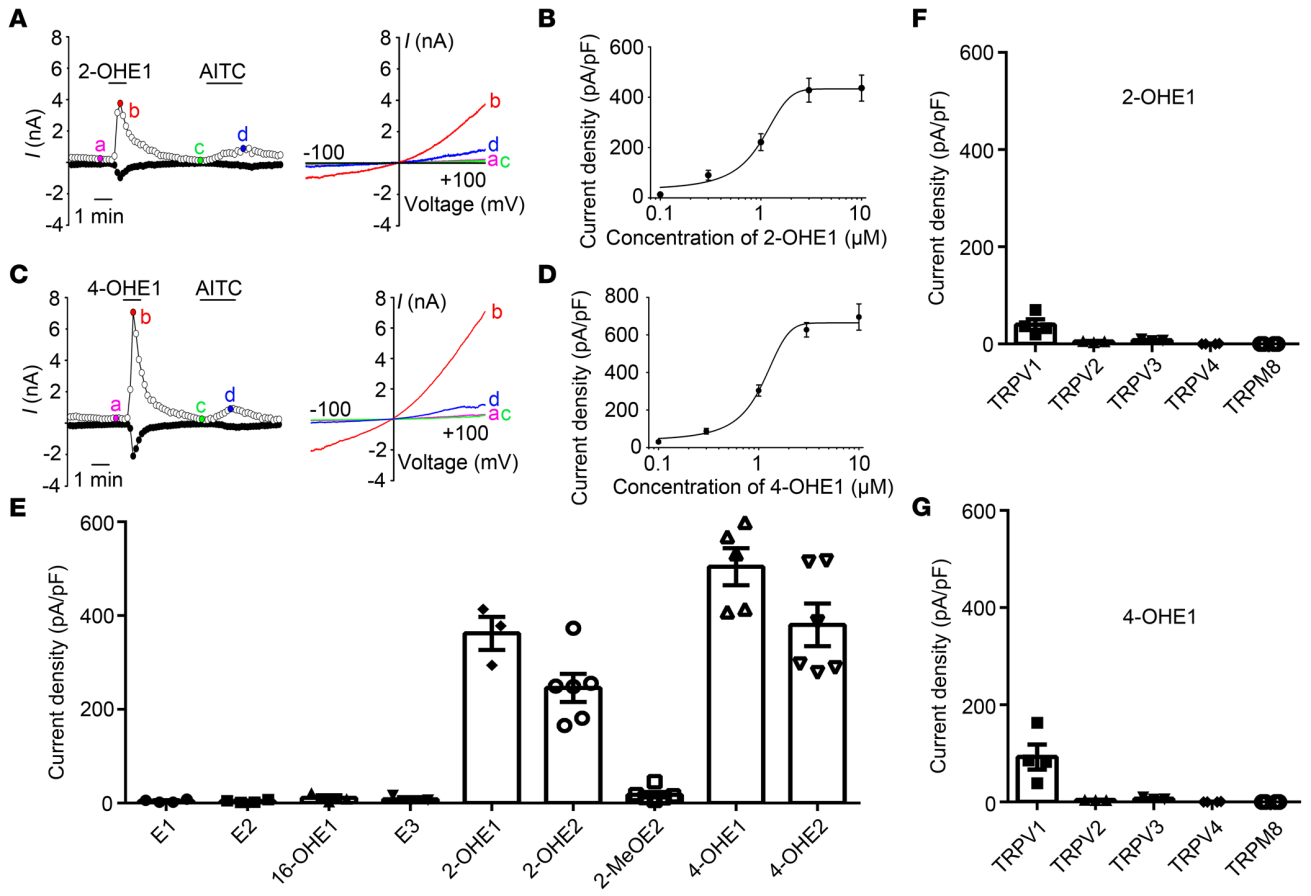
2- and 4-HEMs activate recombinant TRPA1 and TRPV1 expressed in HEK293T cells. (**A**) Representative current traces (left) and I–V curves (right) of TRPA1-dependent currents activated by 2-OHE1 (10 M) and AITC (100 M). (**B**) Concentration-current density curve of 2-OHE1–activated membrane current in TRPA1-expressing HEK293T cells. The EC_50_ = 0.97 ± 0.10 μM. *n =* 5. (**C**) Representative current traces (left) and I–V curves (right) of TRPA1-dependent currents induced by 10 M 4-OHE1 and 100 M AITC. (**D**) Concentration-current density curve of 4-OHE1–induced membrane current in TRPA1-expressing HEK293T cells. The EC_50_ = 1.07 ± 0.09 μM. *n =* 5. (**E**) Bar charts illustrate whole-cell current densities produced by estrogen and its metabolites in TRPA1-expressing HEK293T cells. All chemicals were applied at 10 μM. *n =* 3 to 6. (**F** and **G**) Whole-cell current densities produced by 10 μM 2-OHE1 (**F**) and 10 μM 4-OHE1 (**G**) in HEK293T cells transfected with TRPV1, TRPV2, TRPV3, TRPV4, and TRPM8 channels. *n =* 3 to 4 from 3 independent experiments.

To further confirm that 2- and 4-HEMs directly activate TRPA1 and TRPV1 channels, we heterologously expressed TRP channels in HEK293T cells and measured channel activities using whole-cell patch-clamp recording. Consistent with the results from DRG neurons, both 2- and 4-HEMs, but not E1, E2, and 16-HEMs, activated TRPA1-dependent whole-cell currents (Figure 4, A–E). In addition, 2- and 4-OHE1 activated the recombinant TRPA1 channel with EC_50_ values of 0.97 ± 0.10 μM and 1.07 ± 0.09 μM, respectively (Figure 4, B and D). Moreover, 10 μM 2- and 4-OHE1 evoked measurable membrane currents in HEK293T cells transfected with TRPV1 but not TRPV2, TRPV3, TRPV4, and TRPM8 channels (Figure 4, F and G). Therefore, both 2- and 4-HEMs constitute a family of TRPA1 and TRPV1 activators with a much higher potency to TRPA1.

### Structural basis for activation of TRPA1 and TRPV1 by 2-HEMs and 4-HEMs.

**Figure 5:**
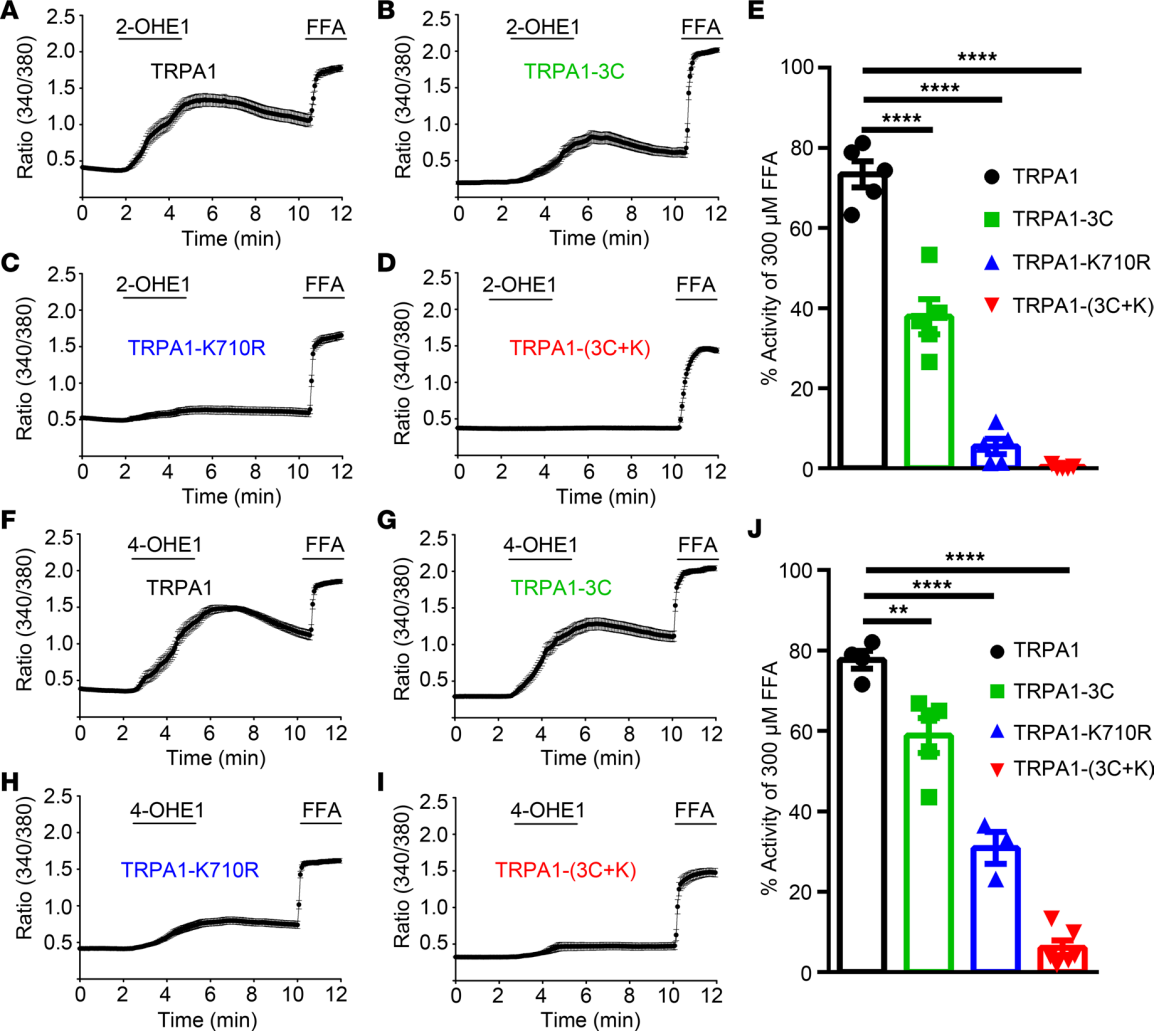
Structural basis of 2-OHE1– and 4-OHE1–induced TRPA1 activation. (**A**–**D**) Averaged time-lapse traces of 2-OHE1–elicited [Ca^2+^]_i_ responses in HEK293T cells transfected with TRPA1 (**A**), TRPA1-3C (**B**), TRPA1-K710R (**C**), and TRPA1-(3C+K) (**D**) constructs; FFA was used as a positive control; (**E**) Quantification of 2-OHE1–induced [Ca^2+^]_i_ responses in HEK293T cells transfected with TRPA1 and its mutants. (**F**–**I**) Averaged time-lapse traces of 4-OHE1–induced [Ca^2+^]_i_ responses in HEK293T cells transfected with TRPA1 (**F**), TRPA1-3C (**G**), TRPA1-K710R (**H**), and TRPA1-(3C+K) (**I**) constructs. (**J**) Quantification of 4-OHE1–induced [Ca^2+^]_i_ responses in HEK293T cells transfected with TRPA1 and its mutants. **P < 0.01, ****P < 0.0001, 1-way ANOVA, *n =* 3 to 6 coverslips per group (>100 cells each) from 3 independent experiments.

Previous studies have shown that electrophiles, such as allyl isothiocyanate (AITC) and 4-hydroxynonenal (HNE), activate TRPA1 through covalent modification of key cysteine and lysine residues within the cytoplasmic domain of the amino terminal (39, 40). To determine whether 2-OHE1 and 4-OHE1 also activate TRPA1 by interacting with these cysteine and lysine residues, we generated TRPA1-3C (a combination of C621S, C641S, and C665S), TRPA1-K710R, and TRPA1-(3C+K710R) mutants that are known to disrupt activation of TRPA1 by known electrophiles using site-directed mutagenesis (40–42). We tested the effect of 2- and 4-HEMs on WT TRPA1 and TRPA1 mutants expressed in HEK293T cells using live-cell Ca^2+^ imaging with a nonreactive TRPA1 agonist, flufenamic acid (FFA), as a positive control (43). We found that HEK293T cells expressing TRPA1-3C or TRPA1-K710R displayed significantly diminished [Ca^2+^]_i_ response induced by either 2-OHE1 or 4-OHE1 when compared with HEK293T cells transfected with WT TRPA1 (Figure 5). Strikingly, the 2- and 4-OHE1–elicited [Ca^2+^]_i_ responses were completely abolished in the HEK293T cells expressing TRPA1-(3C+K710R) mutant (Figure 5), suggesting that 2-OHE1 and 4-OHE1 directly activate TRPA1 through covalent modifications of key cysteine and lysine residues.

Many TRPV1 activators, including capsaicin and resiniferatoxin, activate the TRPV1 channel by interacting with the vanilloid-binding pocket in the cytosolic side, especially Y512 and S513 that specify sensitivity to vanilloid ligands (44–46). We thus examined whether these 2 vanilloid-sensitive TRPV1 residues are involved in the activation of TRPV1 by 2- and 4-OHE1. Indeed, 2- and 4-OHE1–induced [Ca^2+^]_i_ responses were markedly reduced in the Y512A mutant and almost completely abolished in the S513Y mutant but not M548L or T551I (Supplemental Figure 6). These results demonstrated that the vanilloid-binding pocket is essential to the interaction between 2- and 4-HEMs and TRPV1.

### Picomolar concentrations of 2- or 4-OHE1 potentiate AITC- but not capsaicin-induced [Ca^2+^]_i_ response in DRG neurons.

**Figure 6:**
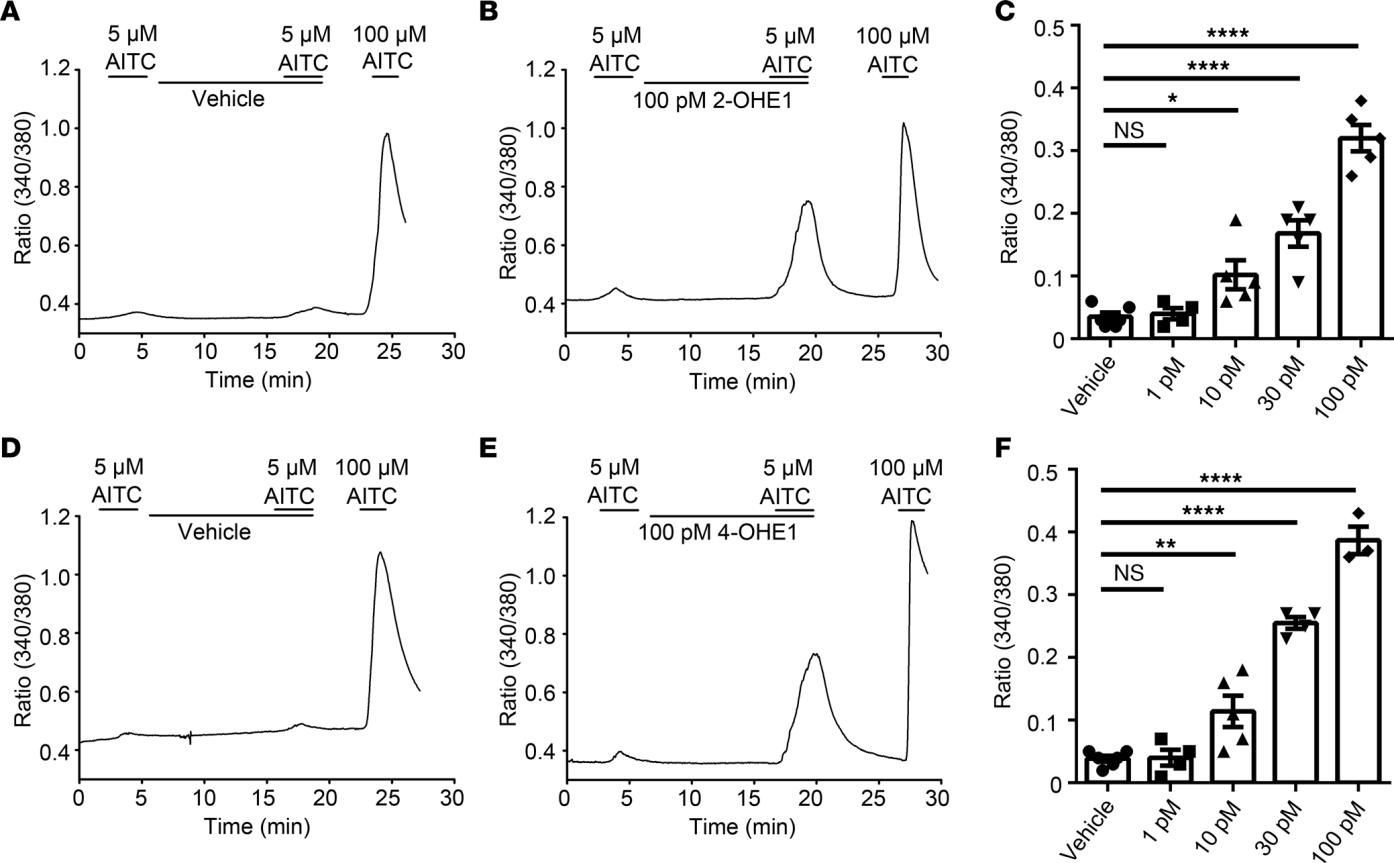
2-OHE1 and 4-OHE1 potentiate AITC-induced [Ca^2+^]_i_ responses in DRG neurons. (**A** and **B**) Preapplication of 100 pM 2-OHE1 (**B**) but not vehicle (**A**) for 10 minutes potentiated 5 μM AITC-induced [Ca^2+^]_i_ responses in DRG neurons isolated from WT mice. (**C**) Bar charts show the potentiating effect of 2-OHE1 on 5 μM AITC-induced [Ca^2+^]_i_ responses in a dose-dependent manner. (**D** and **E**) Effects of vehicle (**D**) and 100 pM 4-OHE1 (**E**) on 5 μM AITC-induced [Ca^2+^]_i_ responses in DRG neurons isolated from WT mice. (**F**) Bar charts illustrate the potentiating effect of 4-OHE1 on 5 μM AITC-induced [Ca^2+^]_i_ responses in a dose-dependent manner. *P < 0.05, **P < 0.01, ****P < 0.0001, 1-way ANOVA, *n =* 3–6 coverslips from at least 3 mice (>200 neurons each).

Sensitization of TRPA1 and TRPV1 is an important mechanism underlying sensory hypersensitivity (47, 48). Therefore, we next tested whether preapplied physiologically relevant concentrations of 2- or 4-OHE1 could sensitize TRPA1 and TRPV1 in DRG neurons. Strikingly, preapplication and coapplication of 10 to 100 pM 2- and 4-OHE1 but not E1 or 16-OHE1 (which were ineffective even at 1 μM) concentration-dependently increased [Ca^2+^]_i_ responses elicited by 5 μM AITC, which otherwise elicited a small [Ca^2+^]_i_ response when applied alone (Figure 6 and Supplemental Figure 7). In marked contrast, pretreatment with 2- or 4-OHE1 did not increase [Ca^2+^]_i_ response induced by capsaicin (Supplemental Figure 8). Combined, these results suggest that 2- and 4-OHE1 sensitize TRPA1 function in primary nociceptors at picomolar concentrations in addition to direct activation of TRPA1 at higher concentrations.

### TRPA1 and TRPV1 are expressed in uterine-innervating DRG neurons.

**Figure 7:**
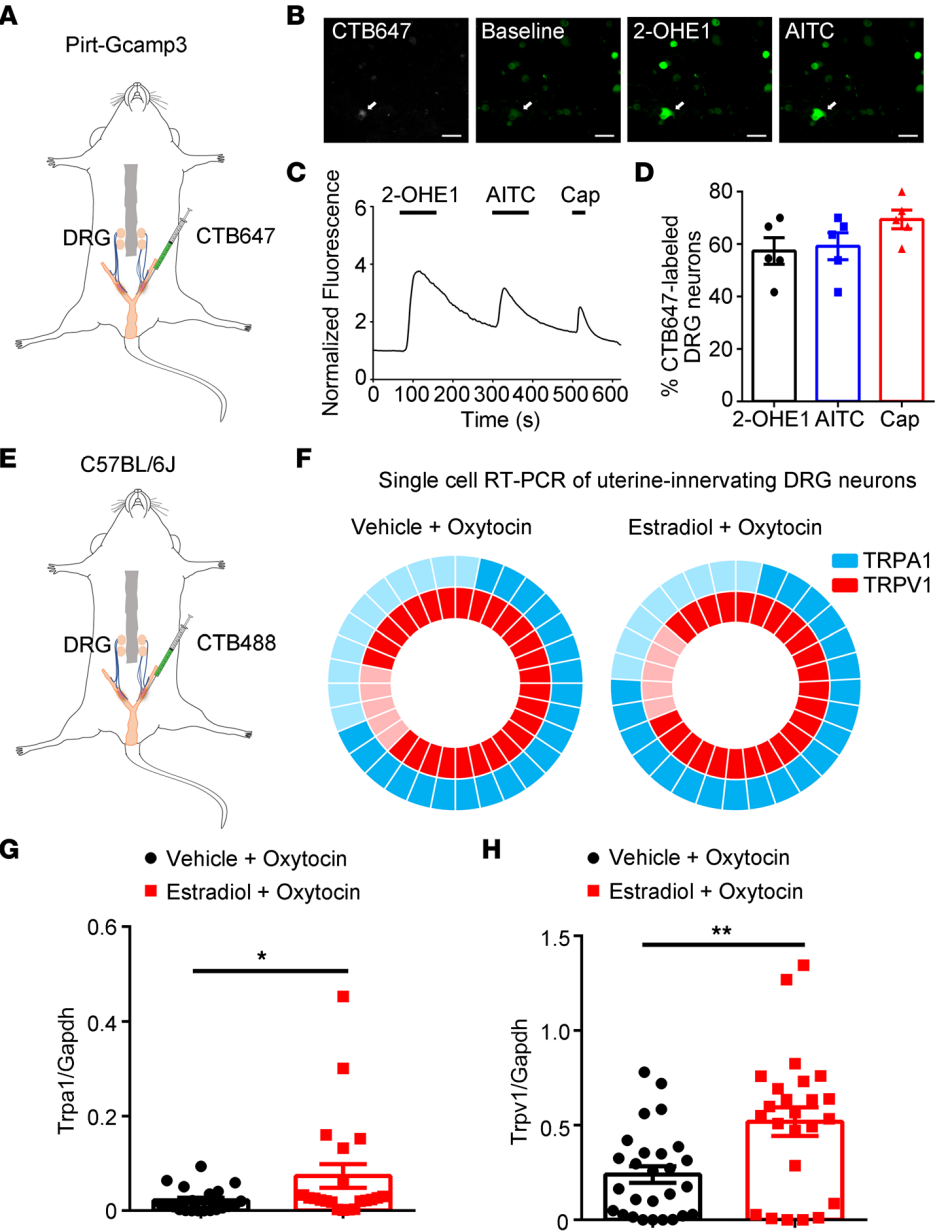
TRPA1 and TRPV1 are expressed in uterine-innervated nociceptors. (**A**) Schematic representation of intrauterine injections of CTB647 into the *Pirt^GCaMP3^* mice. (**B**) Representative fluorescence images of cultured DRG neurons show that 2-OHE1 and AITC evoked [Ca^2+^]_i_ responses in a CTB647-labeled neuron. Scale bar: 50 μm. (**C**) Representative time-lapse trace of 2-OHE1–induced [Ca^2+^]_i_ response in the CTB647-labeled DRG neuron. (**D**) Percentage of CTB647-labeled DRG neurons responding to 2-OHE1, AITC, and capsaicin (Cap). *n =* 5 coverslips from 3 mice. (**E**) Schematic representation of intrauterine injections of CTB488 into the C57BL/6J mice. (**F**) Donut plots showing expression and coexpression of TRPA1 and TRPV1 in 32 individual retrogradely traced colon-innervating DRG neurons from control mice (left) and in 29 individual retrogradely traced colon-innervating DRG neurons from the uterine pain group (right). *n =* 32 cells from 4 mice for control group and *n =* 29 cells from 4 mice for uterine pain group. (**G**) Statistical data of single-cell qRT-PCR in CTB647-labeled neurons show the increased expression of TRPA1 in uterine pain model mice compared with control mice. Unpaired *t* test, *P < 0.05. *n =* 21 cells from 4 mice for vehicle + oxytocin group and *n =* 21 cells from 4 mice for estradiol + oxytocin group. (**H**) Statistical data of single-cell qRT-PCR in CTB647-labeled neurons show the increased expression of TRPV1 in uterine pain model mice compared with control mice. Unpaired *t* test, **P < 0.01. *n =* 27 cells from 4 mice for vehicle + oxytocin group and *n =* 24 cells from 4 mice for estradiol + oxytocin group.

To determine whether DRG neurons innervating the uterus can be activated by HEMs, we injected CTB647 into the uterine wall of *Pirt^GCaMP3^* mice (Figure 7A) and isolated DRG neurons 5 days after injections. We did Ca^2+^ imaging and found 2-OHE1 could also activate CTB647-labeled DRG neurons (Figure 7, B–D). Since prior studies showed increased expression of TRP channels by estrogen treatment (49), we next tested whether chronic treatment of estradiol in a uterine pain model alters the expression of TRPA1 and TRPV1 using single-cell qRT-PCR on CTB488-labeled DRG neurons (Figure 7, E and F). The results showed that in control mice (vehicle + oxytocin), TRPA1 mRNA transcripts were detected in 65.6% (21 of 32) and TRPV1 mRNA transcripts were detected in 84.4% (27 of 32) of CTB488-labeled DRG neurons (Figure 7F). Also, 90.5% (19 of 21) of the TRPA1-positive CTB488-labeled DRG neurons expressed TRPV1 mRNA transcripts. In uterine pain model mice, TRPA1 mRNA transcripts were detected in 72.4% (21 of 29) and TRPV1 mRNA transcripts were detected in 82.8% (24 of 29) of CTB488-labeled DRG neurons (Figure 7F) (estradiol + oxytocin). There was also 90.5% (19 of 21) of the TRPA1-positive CTB488-labeled DRG neurons that expressed TRPV1 mRNA transcripts. More importantly, both mRNA transcript levels of mTRPA1 and TRPV1 were significantly increased in uterus-innervating DRG neurons isolated from uterine pain model mice when compared with those from control mice (Figure 7, G and H).

### TRPA1 mediates 2-OHE1– and 4-OHE1–induced acute mechanical nociception.

**Figure 8:**
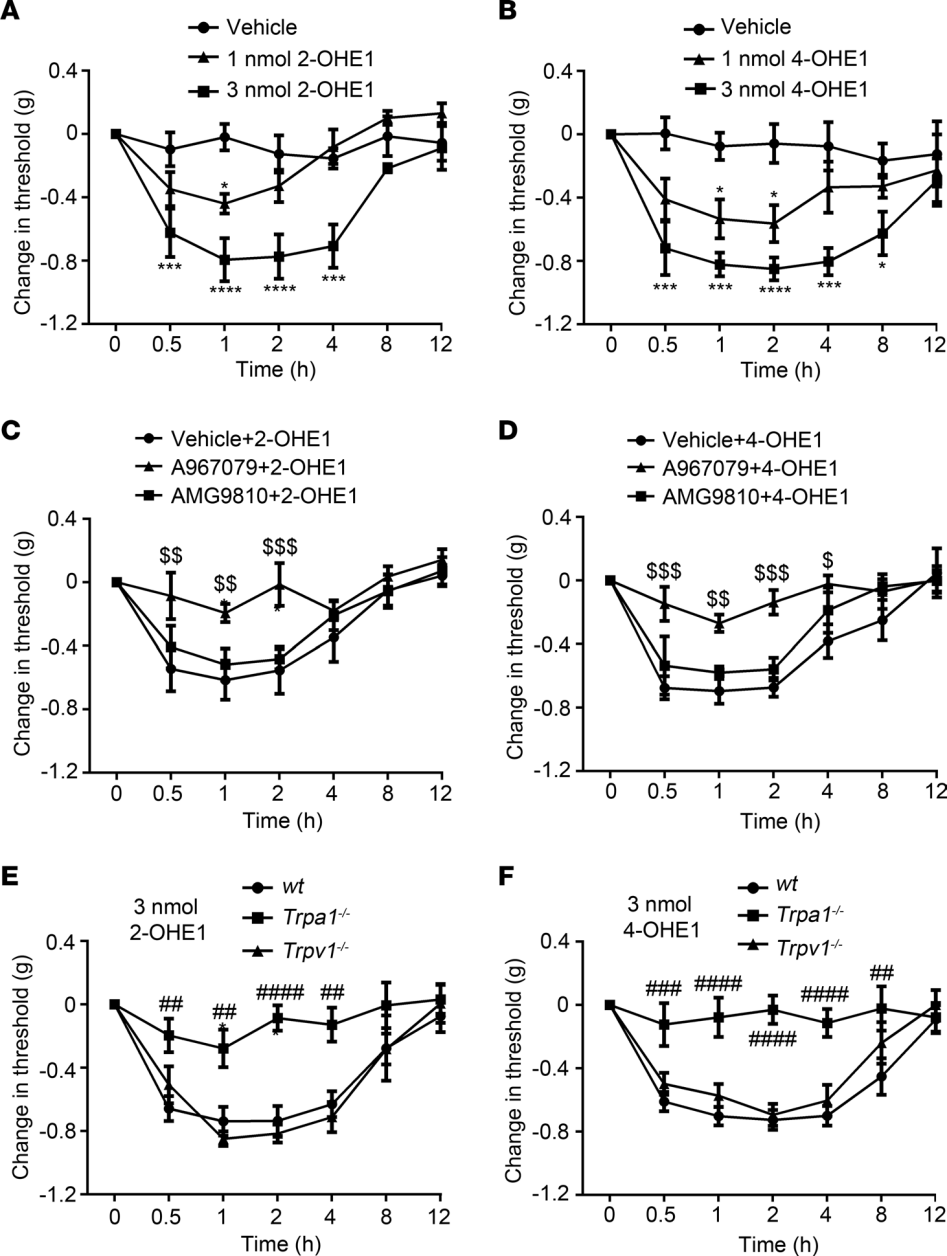
Intraplantar injections of 2-OHE1 and 4-OHE1 produce TRPA1-dependent mechanical allodynia in ovariectomized female mice implanted with estrogen pellets. (**A** and **B**) Intraplantar administration of 2-OHE1 (**A**) and 4-OHE1 (**B**) induced mechanical allodynia in ovariectomized WT female mice implanted with estrogen pellets in a dose-dependent manner. *P < 0.05, ***P < 0.001, ****P < 0.0001, 2-way ANOVA, *n =* 5. (**C** and **D**) TRPA1-specific inhibitor A967079 but not TRPV1-specific inhibitor AMG9810 reverted mechanical allodynia induced by 3 nmol 2-OHE1 (**C**) or 4-OHE1 (**D**). ^$^*P <* 0.05, ^$$^*P* < 0.01, ^$$$^*P <* 0.001, 2-way ANOVA, *n =* 5. (**E** and **F**) Mechanical allodynia induced by 3 nmol 2-OHE1 (**E**) and 4-OHE1 (**F**) was markedly reduced in *Trpa1^−/−^* but not *Trpv1^−/−^* mice. ^##^*P <* 0.01, ^###^*P <* 0.001, ^####^*P <* 0.0001, 2-way ANOVA with Bonferroni’s post hoc analysis, *n =* 5.

We next tested whether estrogen metabolites can directly produce acute nociception through activating TRPA1. Indeed, intraplantar injections of 2-OHE1 and 4-OHE1 but not E1 or 16-OHE1 provoked a robust mechanical pain response in a dose-dependent manner (Figure 8, A and B, and Supplemental Figure 9), which lasted for more than 4 hours. Furthermore, mechanical allodynia, induced by intraplantar injections of 2-OHE1 or 4-OHE1, was significantly reduced by both pharmacological inhibition and genetic ablation of TRPA1 but not TRPV1 function (Figure 8, C–F), suggesting that acute applications of 2- and 4-HEMs directly evoked mechanical hypersensitivity in a TRPA1-dependent but not TRPV1-dependent manner.

### TRPA1 mediates uterine pain caused by chronic estrogen treatment.

**Figure 9:**
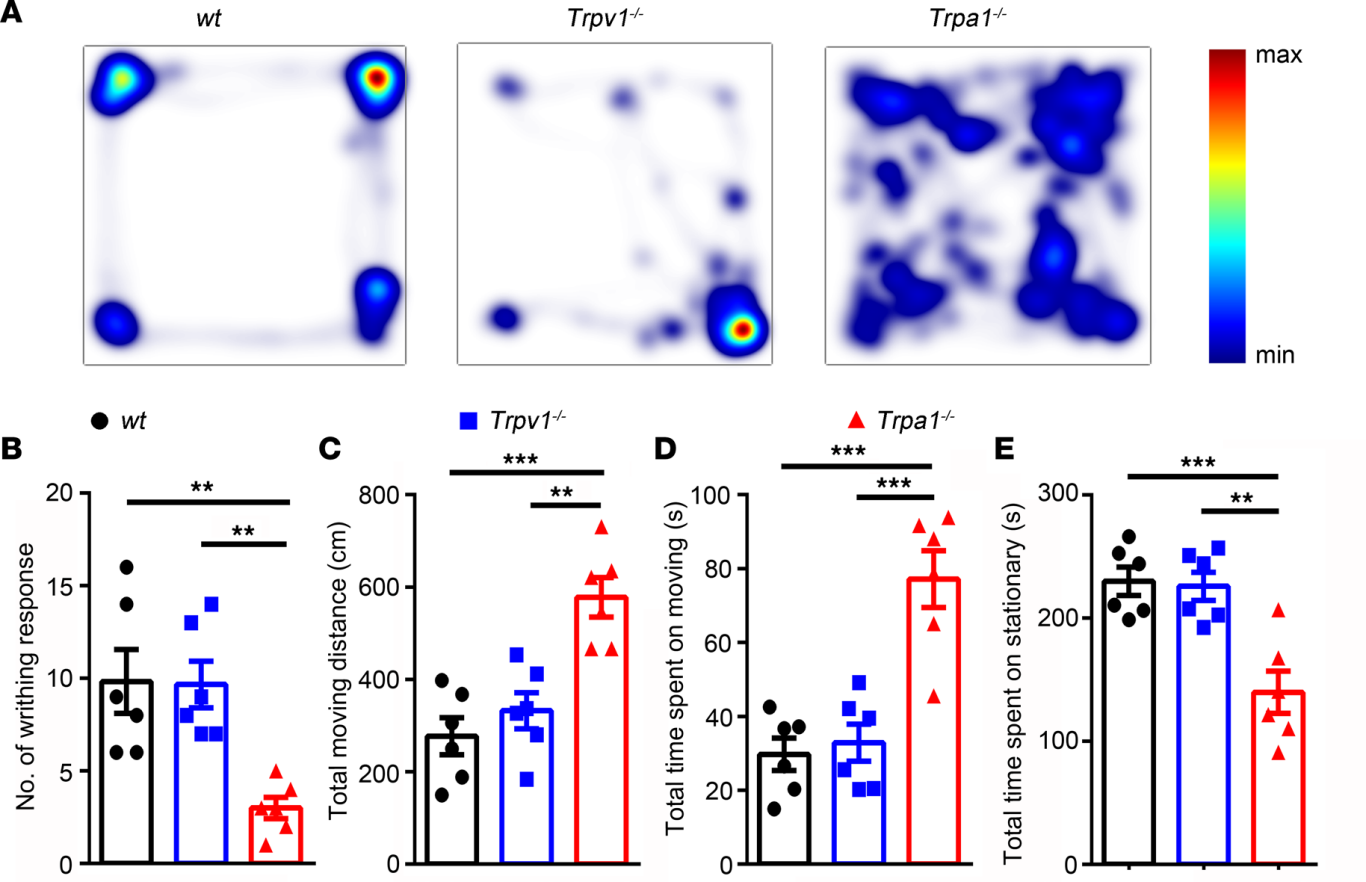
Genetic ablation of TRPA1 but not TRPV1 significantly inhibits pain-related behaviors in a mouse model of uterine pain induced by estrogen in ovariectomized female mice implanted with estrogen pellets. (**A**) Representative images illustrate heatmaps of voluntary movements of WT, *Trpv1^–/–^*, and *Trpa1^–/–^* mice subjected to 3 days of estrogen treatment followed by oxytocin application. (**B**–**E**) Statistical data show the number of writhing responses (**B**), total moving distance (**C**), total time spent moving (**D**), and total time spent stationary (**E**) in WT, *Trpa1^–/–^,* and *Trpv1^–/–^* mice subjected to 3 days of estrogen treatment followed by oxytocin application. **P < 0.01, ***P < 0.001, *n =* 6, 1-way ANOVA.

**Figure 10:**
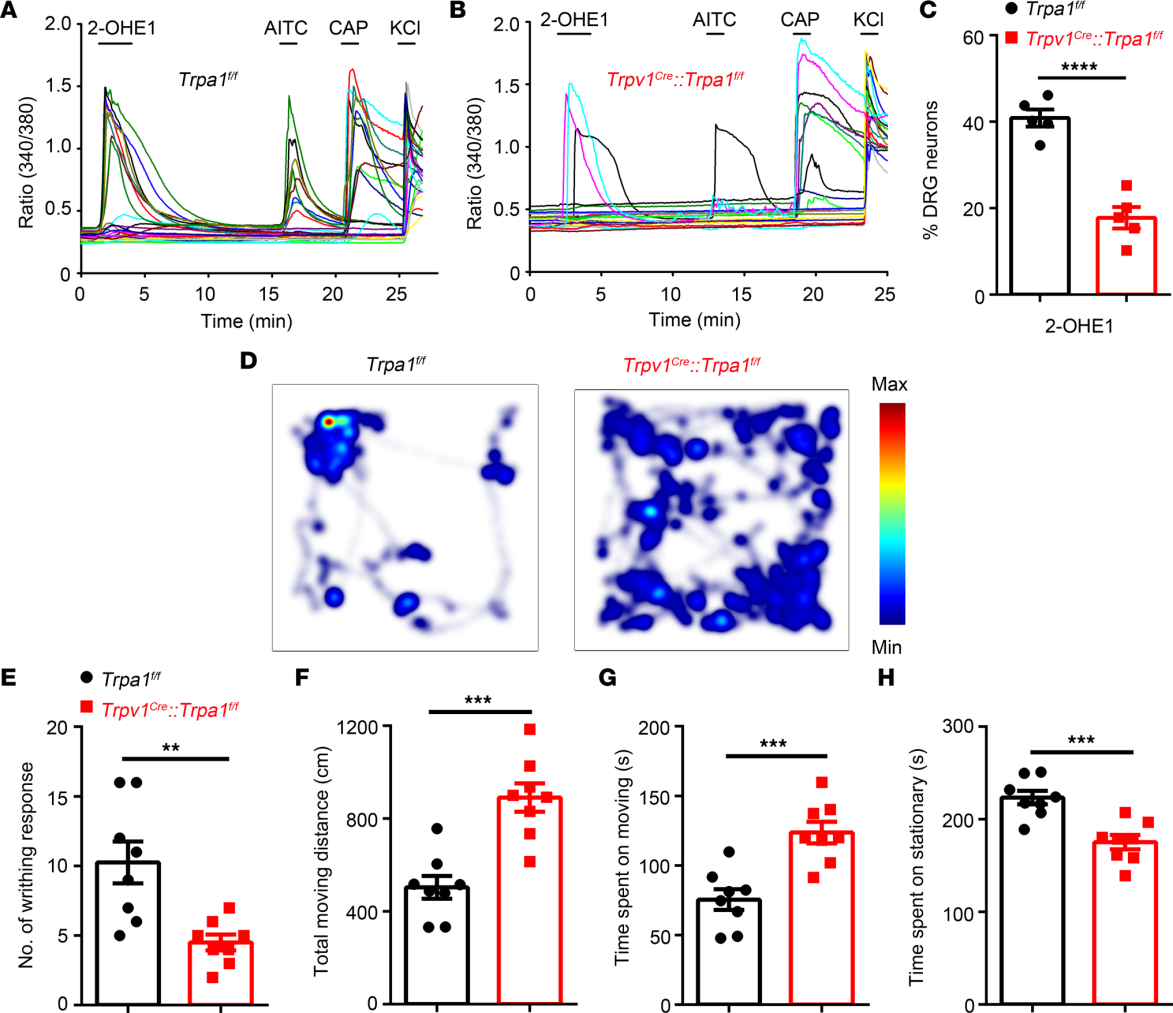
Conditional KO TRPA1 from TRPV1-positive nociceptors significantly inhibits pain-related behaviors in a mouse model of uterine pain. (**A** and **B**) Representative time-lapse trace of 2-OHE1–induced [Ca^2+^]_i_ response in DRG neurons isolated from *Trpa1^fl/fl^* mice (**A**) and *Trpv1^Cre^ Trpa1^fl/fl^* mice (**B**). (**C**) Percentage of DRG neurons responding to 2-OHE1 in DRG neurons isolated from *Trpa1^fl/fl^* mice and *Trpv1^Cre^ Trpa1^fl/fl^* mice. *n =* 5 coverslips from 3 mice per group. ****P < 0.0001, unpaired *t* test. (**D**) Representative images illustrate heatmaps of voluntary movements of *Trpa1^fl/fl^* (left) and *Trpv1^Cre^ Trpa1^fl/fl^* mice (right) subjected to 3 days of estrogen treatment followed by oxytocin treatment. (**E**–**H**) Statistical data show the number of writhing responses (**E**), total moving distance (**F**), total time spent moving (**G**), and total time spent stationary (**H**) in *Trpa1^fl/fl^* and *Trpv1^Cre^ Trpa1^fl/fl^* mice subjected to 3 days of estrogen treatment followed by oxytocin treatment. *n =* 8 mice per group. **P < 0.01, ***P < 0.001, unpaired *t* test.

We showed that TRPA1 was directly activated/sensitized by 2- and 4-HEMs and mediated acute nociception evoked by 2- and 4-OHE1, and previous studies also demonstrated that nociceptor-expressed TRPA1 and TRPV1 mediate both neurogenic inflammation and pain (24, 50). We therefore investigated whether TRPA1 and/or TRPV1 channels are involved in the mouse model of uterine pain induced by chronic estrogen treatment. We measured writhing responses and voluntary movements in the WT *C*57BL/6J mice and their congenic *Trpa1^–/–^* and *Trpv1^–/–^* mice (Figure 9). The total moving distance and the total time spent moving were significantly increased, whereas the number of writhing responses and the time spent stationary were significantly decreased in the *Trpa1^–/–^* but not in *Trpv1^–/–^* mice compared with WT mice (Figure 9), suggesting that TRPA1 but not TRPV1 was required for pain-related responses in this mouse model of uterine pain. We also generated nociceptor-specific *Trpa1* conditional KO mice by crossing *Trpv1^Cre^* mice with *Trpa1^fl/fl^* mice. To confirm the successful ablation of TRPA1 from TRPV1-positive nociceptors, we isolated DRG neurons from *Trpa1^fl/fl^* control littermates and *Trpv1^Cre^* *Trpa1^fl/fl^* mice and tested the effect of 2-OHE1 on isolated DRG neurons (Figure 10, A–C). The 2-OHE1–evoked [Ca^2+^]_i_ response was significantly reduced in DRG neurons isolated from *Trpv1^Cre^* *Trpa1^fl/fl^* mice compared with those isolated from *Trpa1^fl/fl^* control littermates (Figure 10C). More importantly, in this mouse model of uterine pain*, Trpv1^Cre^* *Trpa1^fl/fl^* mice showed significantly reduced pain-related behaviors compared with *Trpa1^fl/fl^* control littermates, recapitulating the phenotype of the global TRPA1-KO mice (Figure 10, D–H). Taken together, these results demonstrated the important role of nociceptor-expressed TRPA1 in the generation of estrogen-induced uterine pain.

### Acute applications of 2-OHE1 and 4-OHE1 elicit uterine pain through TRPA1.

**Figure 11:**
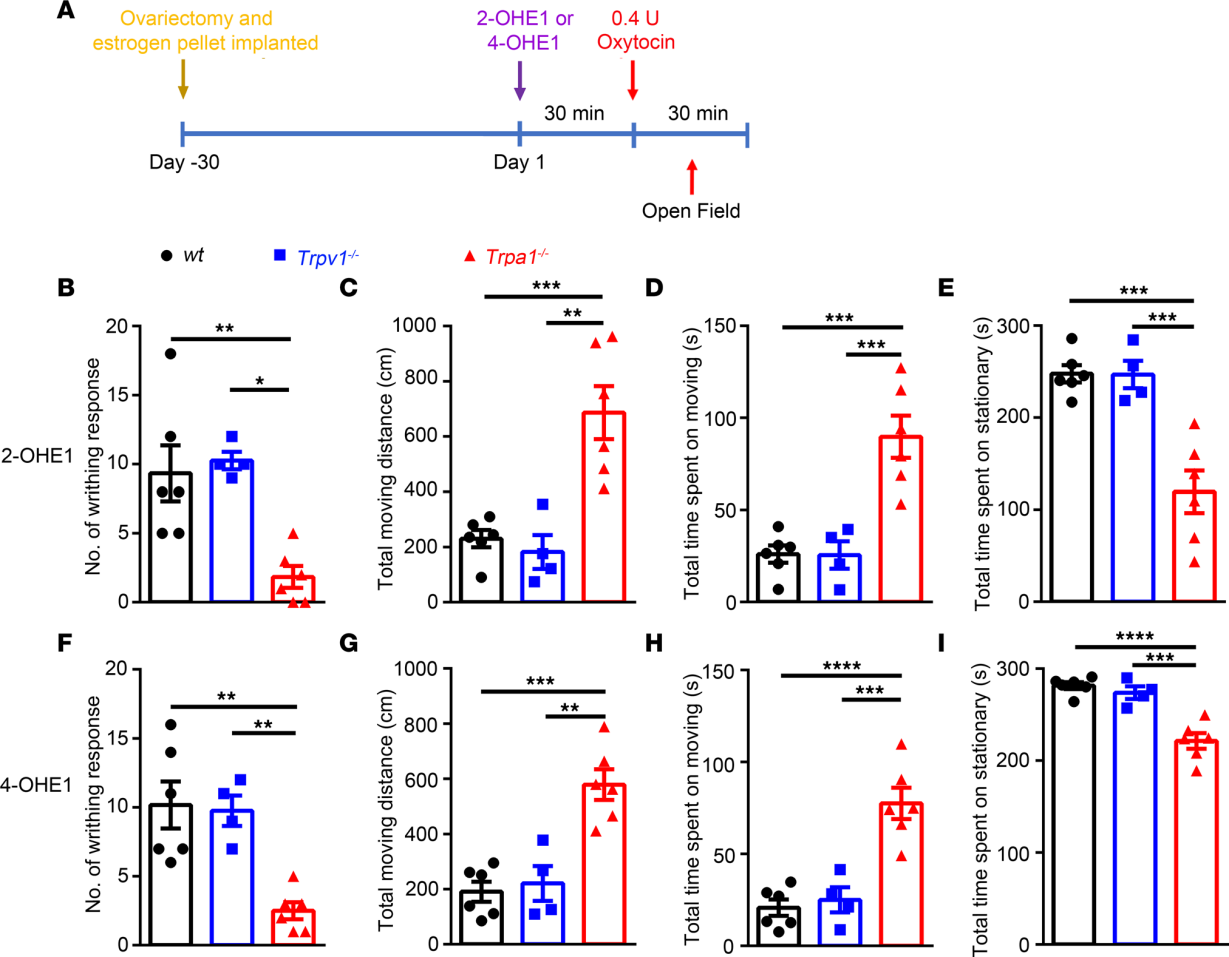
Acute administration of 3 nmol 2- or 4-OHE1 for 30 minutes followed by oxytocin application recapitulates pain-related behaviors in a mouse model of uterine pain induced by 3 days of estrogen treatment. (**A**) Schematic diagram illustrates the protocol for acute i.p. injection of 2-OHE1 and 4-OHE1 to elicit uterine pain in ovariectomized female mice implanted with estrogen pellets. (**B**–**E**) Quantification of writhing responses (**B**), total moving distance (**C**), total time spent moving (**D**), and total time spent stationary (**E**) after i.p. injection of 3 nmol 2-OHE1 for 30 minutes followed by oxytocin application in WT, *Trpa1^−/−^*, and *Trpv1^−/−^* mice. (**F**–**I**) Quantification of writhing responses (**F**), total moving distance (**G**), total time spent moving (**H**), and total time spent stationary (**I**) after i.p. injection of 3 nmol 4-OHE1 for 30 minutes followed by oxytocin application in WT, *Trpa1^−/−^*, and *Trpv1^−/−^* mice. **P < 0.01, ***P < 0.001, ****P < 0.0001, *n =* 4–6, 1-way ANOVA.

We showed that 2- and 4-HEMs directly activate the TRPA1 channel at high concentrations and sensitize TRPA1 function at picomolar concentrations. More importantly, acute application of 2-OHE1 and 4-OHE1 could elicit acute mechanical nociception in a TRPA1 dependent-manner, suggesting that 2- and 4-HEMs may be the cause of the visceral pain behavior in the mouse model of uterine pain generated by chronic estrogen treatment. To test this possibility, we modified the protocol of the uterine pain model and treated the mice acutely with 2- and 4-OHE1 for 30 minutes instead of treating mice with estrogen for 3 days. Strikingly, acute treatment of 2- and 4-OHE1 but not E1 or 16-OHE1 for 30 minutes followed by oxytocin application induced a robust writhing response, which was significantly diminished in the *Trpa1^–/–^* but not *Trpv1^–/–^* mice (Figure 11, A–E, and Supplemental Figure 10). The decreased total moving distance and time spent moving and increased time spent stationary after treatment of 2- and 4-OHE1 followed by oxytocin application were also reversed in the *Trpa1^–/–^* but not *Trpv1^–/–^* mice (Figure 11, B–I), suggesting acute application of 2- or 4-OHE1 was sufficient to sensitize TRPA1 and cause uterine pain in female mice.

## Discussion

A thorough understanding of the cellular and molecular mechanisms accounting for pain processing by estrogen is critical because it offers an opportunity to provide pain relief and personalized pain therapy for estrogen-related pain. In this study, we showed that nociceptor-expressing TRPA1 was critically involved in pain-related behaviors in a mouse model of uterine pain, potentially through a 2- and 4-HEM–mediated sensitization mechanism. First, live-cell Ca^2+^ imaging and whole-cell patch-clamp recordings showed that 2- and 4- HEMs directly activated DRG neurons through TRPA1 and TRPV1 channels in a concentration-dependent manner. Second, at picomolar concentrations, 2- and 4-OHE1 potentiated TRPA1 but not TRPV1 function. Third, intraplantar injections of 2- and 4-OHE1, but not estrogen, evoked acute pain responses in vivo in a TRPA1-dependent manner. Lastly, acute application of 2- and 4-OHE1 was sufficient to produce uterine pain in female mice through TRPA1. Our findings provide convincing evidence that estrogen metabolites can directly activate/sensitize the TRPA1-expressing nociceptors, advancing the understanding of the role of primary nociceptors in estrogen-induced pain and/or enhancement of pain perception in females.

Prior studies have demonstrated that TRP channel expression is regulated by estrogen signaling at both transcriptional and translational levels. For instance, exogenously applied estrogen can regulate the expression of several nociceptive ion channels, including TRPA1 and TRPV1, in nociceptors and non-neuronal cells (21, 51–55). Corroborating these findings, our retrograde labeling experiments also showed that the uterus-innervating nociceptors expressed TRPA1 and TRPV1, and both expression levels were increased in the mouse model of uterine pain induced by chronic estrogen treatment. Our behavioral results support the dominant role of nociceptor-expressed TRPA1 in mediating the estrogen-induced uterine pain-like behaviors: genetic ablation of TRPA1 function in TRPV1-lineage neurons markedly reduced estrogen-induced responses and recapitulated the phenotype seen in the global TRPA1-KO mice.

Surprisingly, we showed that estrogen metabolites but not estrogen itself directly activated TRPA1 and TRPV1 channels and promoted nociceptor excitability. Consistent with our findings, Ma et al. also reported that catechol estrogens stimulate insulin secretion in pancreatic β cells through TRPA1 activation by HEMs at micromolar concentrations (56). Interestingly, although both TRPA1 and TRPV1 are direct targets of 2- and 4-HEMs, only TRPA1 mediates the visceral hypersensitivity caused by estrogen-induced sensitization in the mouse model of uterine pain and acute mechanical nociception evoked by either 2- or 4-OHE1. This discrepancy may result from the lower efficacy and efficiency of HEM activation of TRPV1, as shown in our whole-cell patch-clamp studies using TRPV1-expressing HEK293T cells.

Furthermore, 2- and 4-HEMs at picomolar concentrations only sensitized TRPA1 but not TRPV1 channels. It should be noted that the peak concentration of estradiol in the blood reaches up to more than 500 pM during the human menstrual cycle and in the mouse uterine pain model (Supplemental Figure 1 and ref. 57). Although the estrogen metabolites derived from estradiol at such low concentrations should not effectively activate TRPA1, our results showed that 10 pM of 2- and 4-HEM pretreatment was sufficient to potentiate AITC-induced [Ca^2+^]_i_ increases in DRG neurons, suggesting that low concentrations of 2- and 4-HEMs in the physiological range might be able to sensitize TRPA1 function in primary nociceptors in vivo.

Although recent exciting studies have significantly advanced our understanding about the importance of estrogen signaling in the CNS and immune system in pain perception (58–65), our studies revealed functions of TRPA1 channel and TRPA1-expressing primary nociceptors in mediating the excitatory actions elicited by 2- and 4-HEMs, suggesting additional molecular and cellular targets for estrogen metabolites in the peripheral nervous system. Identification of primary nociceptors as a critical player in estrogen-related pain sensitivity will fill a critical void in understanding how nociceptive TRP channels, especially TRPA1, detect estrogen metabolism and translate it into increased sensory hypersensitivity. These studies should also offer potentially new insights into the development of effective and safe personalized medicines for estrogen-related pain perception. Specifically, rationale-based therapies can be developed to reduce female-dominant pain by targeting TRPA1 signaling, which should avoid the deleterious side effects of current pain medicines. Furthermore, blocking TRPA1 to attenuate estrogen-induced pain sensitization can diminish unwanted, irritating side effects produced by estrogen replacement therapy without compromising the beneficial effects mediated by nuclear estrogen receptor signaling.

## Methods

### Mice.

Adult female C57BL/6J mice (stock no. 000664) used in this study were purchased from The Jackson Laboratory. *Trpa1^+/+^* and congenic *Trpa1^−/−^* mice (stock no. 006401) and *Trpv1^+/+^* and congenic *Trpv1^−/−^* mice (stock no. 003770) on the C57BL/6J background were also obtained from The Jackson Laboratory. *Trpv1^−/−^* and *Trpa1^−/−^* mice were continuously backcrossed to C57BL/6J mice for more than 10 generations. *Trpv1^−/−^* and *Trpa1^−/−^* mice were crossed to generate *Trpa1^−/−^/Trpv1^−/−^* double KO mice. Body weight– and gender-matched WT, *Trpa1^−/−^*, *Trpv1^−/−^*, and *Trpa1^−/−^Trpv1^−/−^* double KO mice were used at about 10 weeks old for all the experiments. *Trpv1^Cre^* mice were donated by Mark Hoon from NIH, and *Trpa1^fl/fl^* mice were provided by Scott Earley from University of Nevada, Reno, Nevada, USA. *Pirt^GCaMP3^* mice were a gift from Xinzhong Dong of Johns Hopkins University, Baltimore, Maryland. All mice were bred at Washington University in Saint Louis, School of Medicine, and housed under a 12-hour light/12-hour dark cycle with food and water provided. All mice were randomly allocated to different experimental groups, and all behavior tests were done by someone who was blinded to the experimental design.

### CTB injection.

Adult female *Pirt^GCaMP3^* or C57BL/6J mice were anesthetized with 4% isoflurane followed by 1.5% isoflurane to maintain anesthesia, and artificial tears were used to prevent dehydration of eyes. Mice were placed on a sterile surgical pad and covered with a sterile surgical drape after shaving and sterilization of the abdomen. To expose the uterine, a midline incision was made through the abdominal wall, and 2 injections of CTB647 or CTB488 were injected bilaterally into the wall of each uterine horn at 100 nL/minute with a pulled glass pipette. After injection, the abdominal wall was sutured, and skin was closed with surgical sutures and antibiotic ointment was applied to the surgical site. Five days after the operation, tissues were collected for analysis.

### Isolation and culture of DRG neurons.

Mouse DRG neurons were isolated and cultured as previously described (42, 66). According to published retrograde tracing results, DRGs between T12 and S2 were isolated (67). Briefly, the spinal column was firstly removed from the mice and then placed in 2 mL ice-cold HBSS. Laminectomies were performed and bilateral T12-S2 DRGs were dissected out. Connective tissues were removed, and DRGs were placed into 1 mL Ca^2+^/Mg^2+^-free HBSS with 2 μL saturated NaHCO_3_, 0.35 mg L-cysteine, and 20 U papain (Worthington Biochemical) and incubated at 37°C for 10 minutes. After centrifugation, the supernatants were removed and DRG was further treated with 1 mL Ca^2+^/Mg^2+^-free HBSS containing 3.75 mg collagenase type II (Worthington Biochemical) and 7.5 mg dispase (Worthington Biochemical) for 15 minutes. Neurons were gently triturated, pelleted, and then resuspended in neurobasal-A culture medium containing 2% B-27 supplement (Thermo Fisher Scientific), 100 U/mL penicillin plus 100 μg/mL streptomycin (Sigma-Aldrich), 100 ng/mL nerve growth factor (Sigma-Aldrich), 20 μg/mL glial cell–derived neurotrophic factor (Sigma-Aldrich), and 10% heat-inactivated FBS (Sigma-Aldrich). After plating, DRG neurons were kept in a humidified incubator at 37°C for at least 24 hours.

### Single DRG neuron picking.

DRG neurons were purified with a 15% BSA density gradient column, and CTB488-labeled neurons were visually identified using a Nikon Eclipse TE200-S microscope. The identified neuron was picked using a micromanipulator (Sutter Instrument). A pulled glass electrode filled with HBSS was used to get individual DRG neurons, and then the neuron was drawn into the tip of glass electrode using negative pressure. The neuron was transferred to a PCR tube containing 10 μL of Single Cell Lysis/Dnase I solution (4458237, Invitrogen) and was processed according to the manual.

### Single-cell qRT-PCR.

Single-cell qRT-PCR was performed with Invitrogen Single Cell-to-CT kit (4458237, Invitrogen) according to the manufacturer’s manual. TaqMan assays were used to measure the abundance of Trpa1 (TaqMan assay Mm01227437_m1), Trpv1 (TaqMan assay Mm01246300_m1), and Gapdh (TaqMan assay Hs02758991_g1).

### HEK293T cell culture and transfection.

HEK293T cells were purchased from ATCC (CRL-3216). Before culturing in the lab, cells were tested for mycoplasma contamination. Cells were cultured in DMEM (Life Technologies) containing 100 U/mL penicillin plus 100 μg/mL streptomycin and 10% FBS (Life Technologies) in a humidified incubator at 37°C with 5% CO_2_.

cDNAs for mouse TRPV1 (mTRPV1), individual mTRPV1 mutants, human TRPA1 (hTRPA1), individual hTRPA1 mutants, mouse TRPV2 (mTRPV2), mouse TRPV3 (mTRPV3), rat TRPV4 (rTRPV4), or mouse TRPM8 (mTRPM8) were transiently transfected to HEK293 cells for at least 24 hours using Lipofectamine 2000 (Invitrogen). QuikChange II XL Mutagenesis kit (Agilent Technologies Inc.) and DNA sequencing were performed to make all the mutants. TRPA1 and TRPV1 mutants are described in a published paper (42).

### Ratiometric measurement of [Ca2+]i.

Before calcium imaging experiments, cultured DRG neurons and HEK293T cells transfected with TRP channels or their mutant plasmid were placed in culture medium containing 4 μM Fura-2 AM (Life Technologies) at 37°C for 60 minutes. Then, cells were washed 3 times and placed in HBSS at room temperature for 30 minutes. During the experiment, fluorescence at 340 nm and 380 nm excitation wavelengths was recorded using an inverted Nikon Ti-E microscope controlled by NIS-Elements imaging software (Nikon Instruments Inc.). Fura-2 ratios (F340/F380) was used to reflect changes in [Ca^2+^]_i_ upon stimulation, and threshold of activation was defined as 3 SD above the average (~20% above the baseline).

### Whole-cell patch-clamp recordings.

Whole-cell patch-clamp recordings were done by an Axon 700B amplifier (Molecular Devices) at room temperature (22°C–24°C) on the stage of an inverted phase-contrast microscope equipped with a filter set for GFP visualization (42, 66). For DRG neurons, small or medium diameter cells (<25 μm diameter) were selected in recording. For transfected HEK293T cells, GFP-expressing cells were selected during recording. Pipettes were pulled using a P-1000 pipette puller from borosilicate glass (Sutter Instrument, BF 150-86-10). The pulled pipettes had resistance of 2 to 5 megaohms. The pipette solution contained 140 mM KCl, 2 mM EGTA, and 10 mM HEPES; pH was adjusted to 7.3 with KOH, and osmolarity was adjusted to 315 mOsm/L with sucrose. The extracellular solution contained 140 mM NaCl, 5 mM KCl, 0.5 mM EGTA, 1 mM MgCl_2_, 10 mM glucose, and 10 mM HEPES (pH was adjusted to 7.4 with NaOH, and the osmolarity was adjusted to ≈340 mOsm/L with sucrose).

### Ovariectomy and 17β-estradiol replacement and assay for 17β-estradiol.

Female mice were ovariectomized bilaterally, and a slow-release pellet of 17β-estradiol (0.1 mg/pellet, 90-day release, Innovative Research of America) was inserted subcutaneously. Serum concentrations of 17β-estradiol were measured at the Ligand Assay and Analysis Core in the Center for Research in Reproduction at the University of Virginia using ELISA kits. The assay sensitivity was 3 pg/mL.

### Mouse model of uterine pain and analysis of voluntary movements.

For the uterine pain mouse model, the ovariectomized and 17β-estradiol–replaced female mice were pretreated with estradiol benzoate (1 mg/kg/day, i.p.) for 3 consecutive days. On the fourth day, mice were i.p. injected with 0.4 U of oxytocin. Immediately after oxytocin injection, the mice were unrestrainedly placed in a 25 cm × 25 cm custom cage, and the voluntary movements of the mice were recorded for 30 minutes. The number of writhing responses within 30 minutes was counted. The distance mice traveled around the cage, the time mice spent moving, and the time mice spent stationary within the first 5 minutes were measured using Ethovision XT tracking software (Noldus). After the recording, the animals were used for mechanical allodynia testing.

### Mechanical allodynia behavioral test.

The mechanical allodynia test was performed as previous described (68, 69). Briefly, each mouse was placed individually in a Plexiglas chamber and acclimated for 1 hour before testing. Paw withdrawal threshold was measured using Von Frey filaments, starting with the 0.4 g filament. Von Frey filaments ranging from 0.02 g to 2 g bending force were applied to the plantar skin of the right hind paw, using the up-down method to determine the threshold. After baseline determination, 10 μL of vehicle or estrogen metabolites were injected intraplanarly in the right hind paw of mice (ipsilateral paw). The mice were then tested for mechanical allodynia over a time course of 12 hours (0.5, 1, 2, 4, 8, and 12 h). AMG9810 (10 mg/kg, i.p. injection) and A967079 (10 mg/kg, i.p. injection) were given 30 minutes before paw injections of 2-OHE1 or 4-OHE1. All experiments were performed blindly with respect to genotype and treatment.

### Statistics.

All retrograde tracing, patch-clamp, and Ca^2+^ imaging experiments were repeated using DRG neurons from at least 3 mice. GraphPad Prism 7.0 was used to perform statistical analysis. Unless stated otherwise, data are presented as mean ± SEM. In general, the exact value of the sample size (*n*) is presented in the figure legends. An unpaired 2-tailed Student’s *t* test with equal variance was used where 2 groups were compared. One-way ANOVA or 2-way ANOVA with Bonferroni’s post hoc analysis was used to calculate *P* values where multiple groups were compared, as figure legends show. *P* values of less than 0.05 were considered significantly different.

### Study approval.

All the animal experimental procedures were done according to the guidelines of the International Association for the Study of Pain and the NIH and were approved by the IACUC of Washington University School of Medicine.

## Author contributions

The manuscript was written through contributions of all authors. All authors have given approval to the final version of the manuscript. ZX, JF, TC, and HH designed the experiments. ZX, JF, and TC performed the experiments and analyzed the data. RM, YW, FL, and SKE assisted with ovariectomy and estrogen pellet implantation. MDE and AD helped in estrogen detection. ZY, YZ, KZ, YY, and XH assisted with behavior assays. QL donated mice. ZX, JF, and HH wrote the manuscript. HH supervised the project. First authorship order position is based on intellectual contribution to design of the study and interpretation of data.

## Supplementary Material

Supplemental data

## Version 1 04/14/2022

In-Press Preview

## Version 2 05/23/2022

Electronic publication
