# Supplementary material for: Estrogen metabolites increase nociceptor hyperactivity in a mouse model of uterine pain
Source: JCI Insight. 2022 May 23;7(10):e149107. doi: 10.1172/jci.insight.149107 (PMC9220826; doi:10.1172/jci.insight.149107)
Supplement: Supplemental data [file jciinsight-7-149107-s065.pdf]

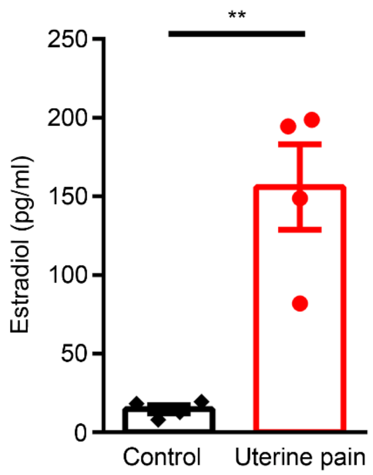

Fig S1. Plasma estradiol concentrations in the control and uterine pain model mice. Pre-application of estradiol benzoate for 3 consecutive days in ovariectomized female mice implanted with estrogen pellets shows higher concentrations of plasma estradiol compared with that in ovariectomized female mice implanted with estrogen pellets treated with vehicle only. **$p<0.01$, $n=4$, unpaired t-test.

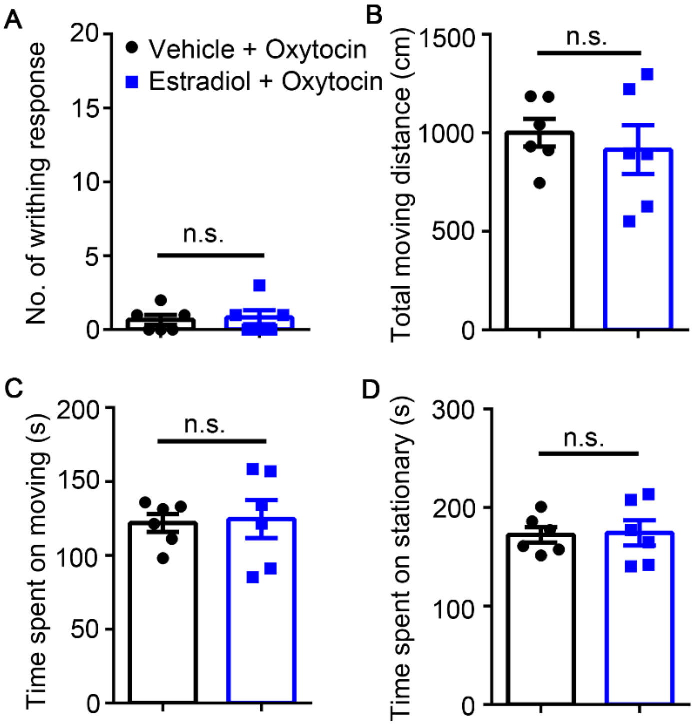

Fig S2 Pretreatment of estradiol benzoate followed by oxytocin application did not induce pain-related responses in male mice. (A) Oxytocin-induced writhing response in "Vehicle + Oxytocin" group and "Estradiol + Oxytocin" group. (B-D) Quantification of voluntary movements including total moving distance (B), total time spent on moving (C) and total time spent on stationary (D), n=6 mice per group, unpaired t-test, n.s. not significant.

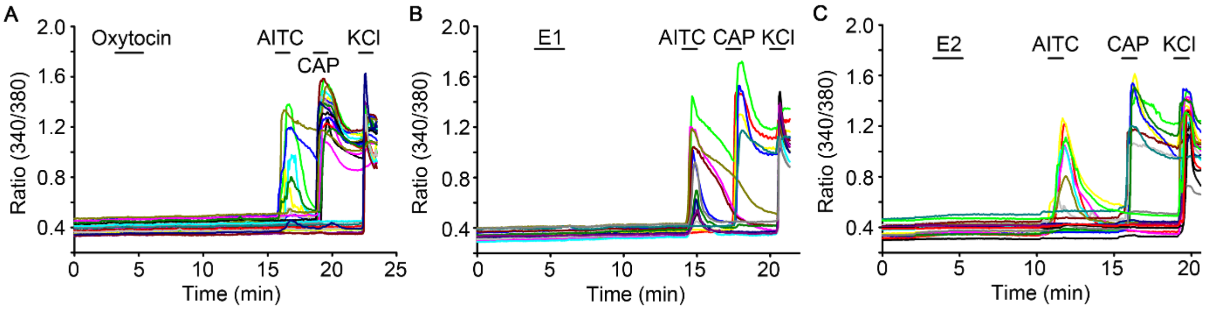

Fig S3. DRG neurons are not activated by application of oxytocin, E1 or E2. (A) Representative time-lapse traces show that 30 μM oxytocin does not induce $[Ca^{2+}]_i$ response in DRG neurons isolated from *wt* mice. (B-C) Representative time-lapse traces show that 100 μM E1 (B) or E2 (C) does not induce $[Ca^{2+}]_i$ response in DRG neurons. One of three independent experiments is shown.

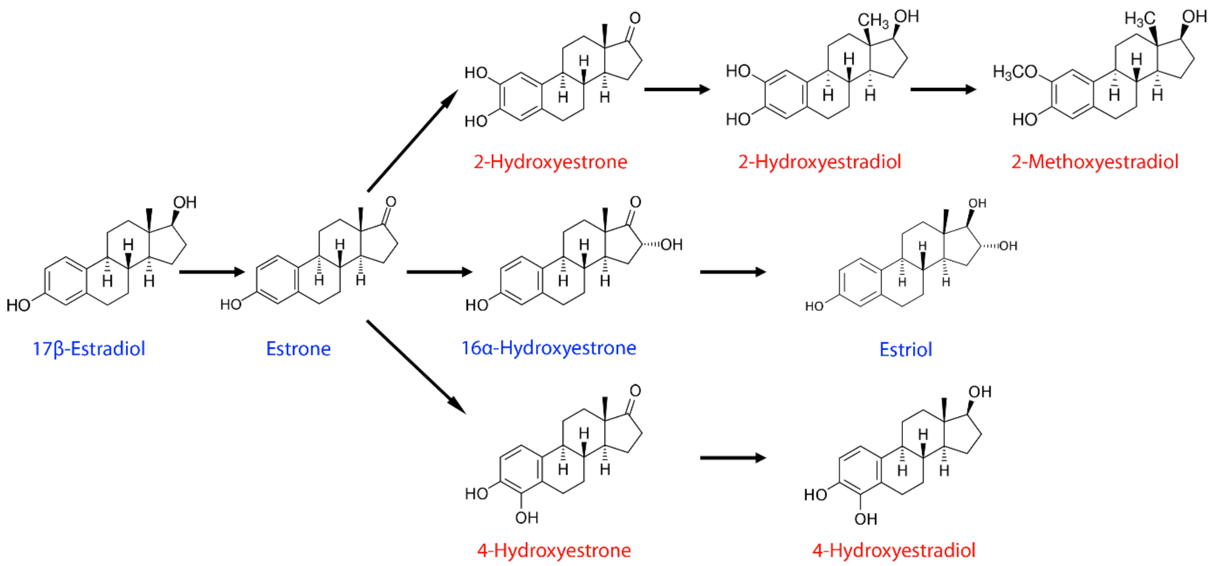

Fig S4. Formation of 2-, 4-, and 16-hydroxylated estrogen metabolites from E1 and E2.

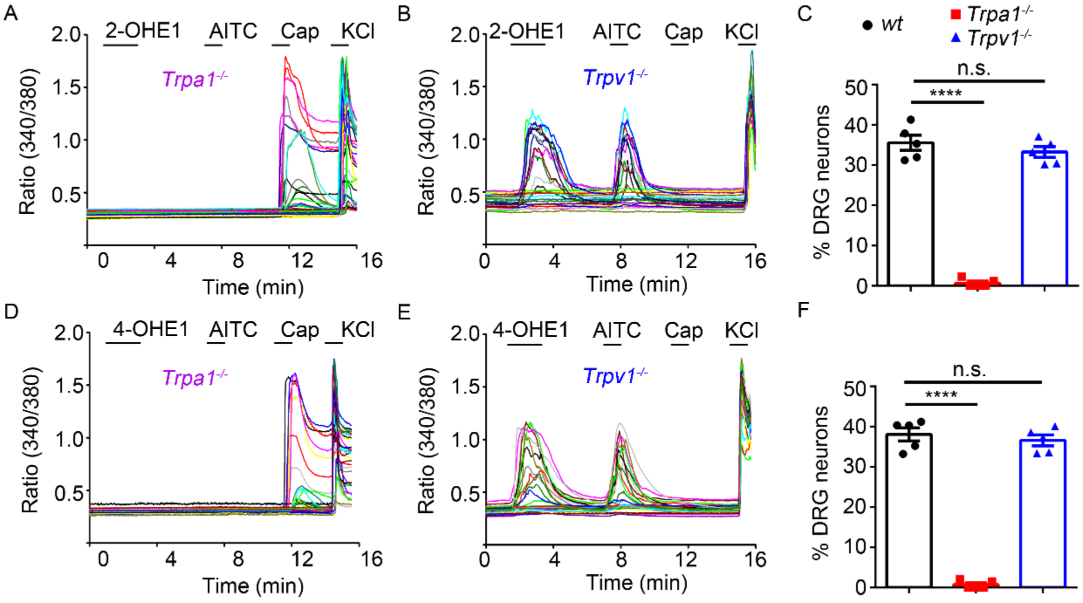

Fig S5. Activation of DRG neurons induced by a low concentration of 2- or 4-OHE1 (10 μM) relies on the function of TRPA1 but not TRPV1. (A-B) Representative time-lapse traces show that 10 μM 2-OHE1 induces $[Ca^{2+}]_i$ response in *Trpv1*$^{-/-}$ (B) but not *Trpa1*$^{-/-}$ (A) DRG neurons. (C) Percentage of 2-OHE1 (10 μM) responding DRG neurons isolated from *wt*, *Trpa1*$^{-/-}$ and *Trpv1*$^{-/-}$ mice. n=5 coverslips from 3 mice per group (>200 neurons each). n.s. not significant, ****$p < 0.0001$, One-way ANOVA. (D-E) Representative time-lapse traces show that 10 μM 4-OHE1 induces $[Ca^{2+}]_i$ response in *Trpv1*$^{-/-}$ (E) but not *Trpa1*$^{-/-}$ (D) DRG neurons. (F) Percentage of 4-OHE1 (10 μM) responding DRG neurons isolated from *wt*, *Trpa1*$^{-/-}$ and *Trpv1*$^{-/-}$ mice. n=4-5 coverslips from 3 mice per group (>200 neurons each). n.s. not significant, ****$p < 0.0001$, One-way ANOVA.

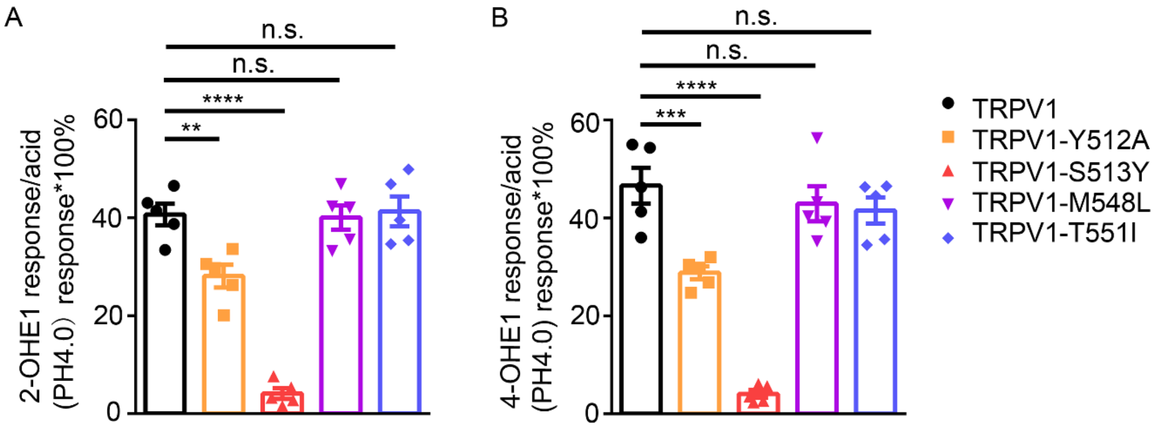

Fig S6. Structural basis of TRPV1 activation by 2-OHE1 and 4-OHE1. (A) Quantification of 2-OHE1-induced $[Ca^{2+}]_i$ response in HEK293T cells transfected with TRPV1 and TRPV1 mutants. (B) Quantification of 4-OHE1-induced $[Ca^{2+}]_i$ response in HEK293T cells transfected with TRPV1 and TRPV1 mutants. All responses were normalized to that elicited by pH 4.0 solution. **P<0.01, ***p < 0.001, ****p < 0.0001, One-way ANOVA, n=5 coverslips per group from three independent experiments.

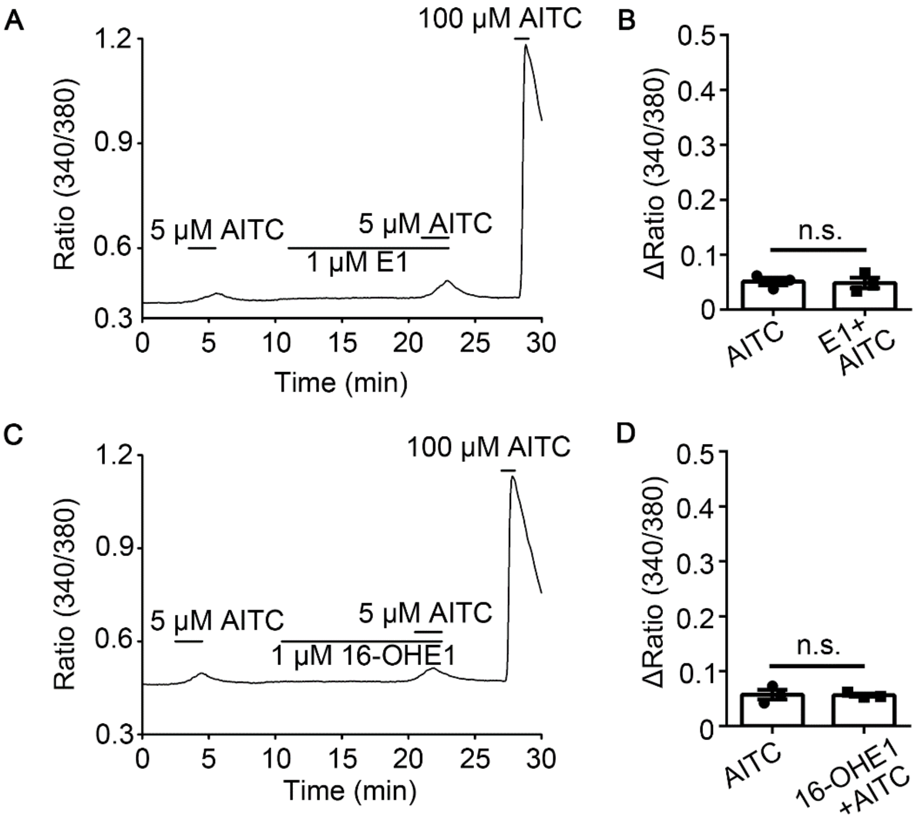

Fig S7. Pre-incubation of E1 and 16-OHE1 has no effect on AITC-induced $[Ca^{2+}]_i$ response in DRG neurons. (A-B) Representative trace (A) and bar charts (B) show that E1 has no effect on 5 μM AITC-induced $[Ca^{2+}]_i$ response compared with vehicle. (C-D) Representative trace (C) and bar charts (D) show that 16-OHE1 has no effect on 5 μM AITC-induced $[Ca^{2+}]_i$ response compared with vehicle. n=6 coverslips from 3 mice per group (>200 neurons each), unpaired t-test, n.s. not significant.

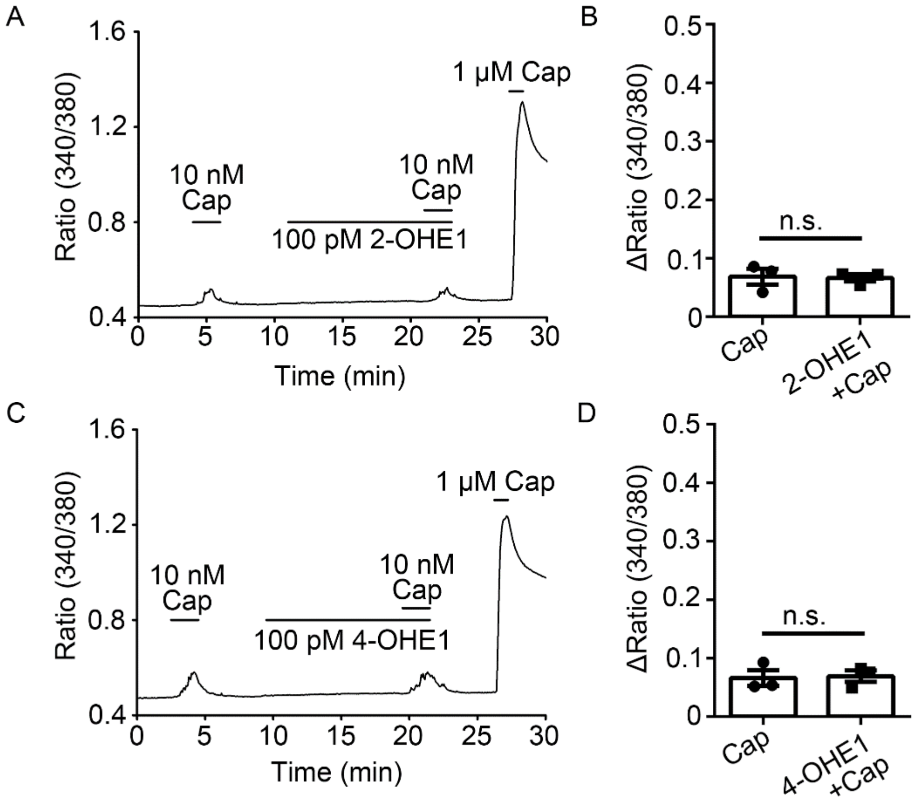

Fig S8. Pre-incubation of 2-OHE1 and 4-OHE1 has no effect on capsaicin-induced $[Ca^{2+}]_i$ response in DRG neurons. (A, B) Representative time-lapse trace (A) and summarized data (B) show that 2-OHE1 does not affect capsaicin-induced $[Ca^{2+}]_i$ response compared with vehicle in DRG neurons isolated from *wt* mice. (C, D) Representative time-lapse trace (C) and summarized data (D) illustrate that 4-OHE1 does not affect capsaicin-induced $[Ca^{2+}]_i$ response compared with vehicle in DRG neurons isolated from *wt* mice. n=5 coverslips from 3 mice per group (>200 neurons each), unpaired t-test, n.s. not significant.

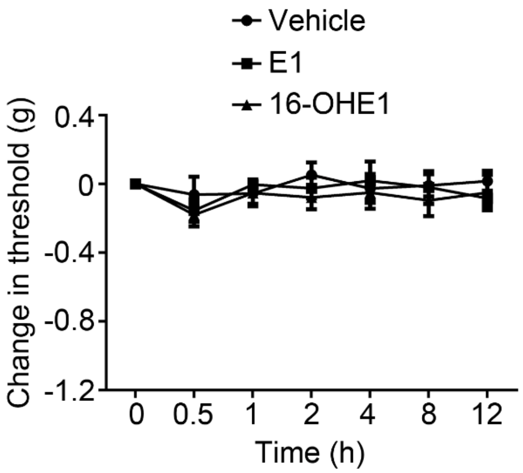

Fig S9. Intraplantar injections of E1 or 16-OHE1 do not induce mechanical allodynia in ovariectomized female mice implanted with estrogen pellets. Changes in mechanical threshold in *wt* mice injected with vehicle, E1 or 16-OHE1. n=5, Two-way ANOVA.

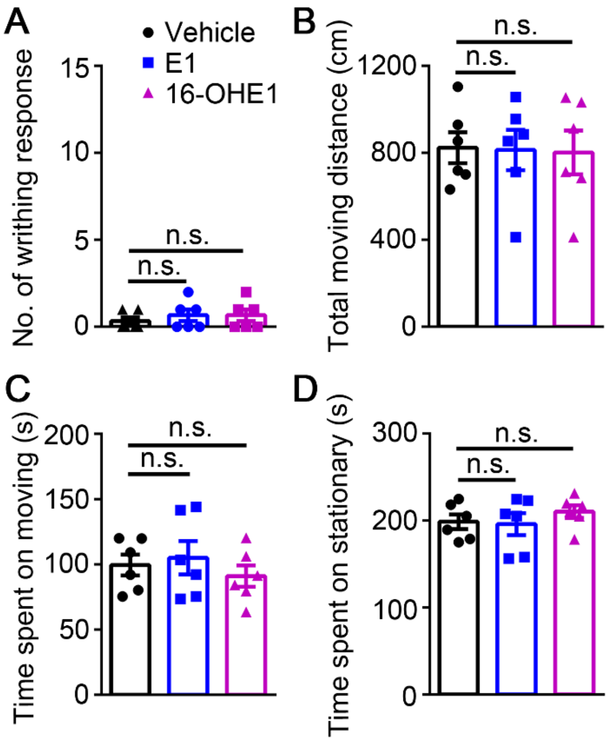

Fig S10. Acute administration of E1 or 16-OHE1 doesn't induce pain-related behaviors in the mouse model of uterine pain. (A-D) Quantification of writhing response (A), total moving distance (B), total time spent on moving (C), and total time spent on stationary (D) after acute application of E1 or 16-OHE1 for 30 min followed by oxytocin application in *wt* mice. n=6, One-way ANOVA, n.s. not significant.
